# Identification of Differentially Expressed microRNAs Associated with Ischemic Stroke by Integrated Bioinformatics Approaches

**DOI:** 10.1155/2022/9264555

**Published:** 2022-10-10

**Authors:** Shengqiang Jiang, Jie Wu, Yan Geng, Yuting Zhang, Yupeng Wang, Jinrong Wu, Chunqu Lu, Guoxuan Luo, Jie Zan, Yong Zhang

**Affiliations:** ^1^The Second School of Clinical Medicine, Southern Medical University, Guangzhou, Guangdong 510515, China; ^2^Department of Neurosurgery, Guangdong Second Provincial General Hospital, Guangzhou, Guangdong 510317, China; ^3^School of Biomedical and Pharmaceutical Sciences, Guangdong University of Technology, Guangzhou 510006, China; ^4^Department of Outpatient, Guangdong Second Provincial General Hospital, Guangzhou, Guangdong 510317, China

## Abstract

Ischemic stroke (IS) is one of the leading causes of disability and mortality worldwide. This study aims to find the crucial exosomal miRNAs associated with IS by using bioinformatics methods, reveal potential biomarkers for IS, and investigate the association between the identified biomarker and immune cell pattern in the peripheral blood of IS patients. In this study, 3 up-regulated miRNAs (hsa-miR-15b-5p, hsa-miR-184, and hsa-miR-16-5p) miRNAs in the serum exosomes between IS patients and healthy controls from GEO database (GSE199942) and 25 down-regulated genes of peripheral blood mononuclear cells of IS patients from GSE22255 were obtained with the help of the R software. GO annotation and KEGG pathway enrichment analysis showed that the 25 down-regulated genes were associated with coenzyme metabolic process and were mainly enriched in the N-glycan biosynthesis pathway. Furthermore, we performed the LASSO algorithm to narrow down the above 25 intersected genes, and identified 8 key genes which had a good diagnostic value in discriminating IS patients from the healthy controls analyzed with ROC curve. CIBERSORT algorithm indicated that the abundance of M0 macrophages and resting mast cells was significantly lower than that of the control group. The spearman correlation analysis showed that STT3A was negatively correlated with the proportion of follicular helper T cells, activated NK cells and resting dendritic cells. Finally, GSE117064 showed that has-miR-16-5p was more advantageous for diagnosing stroke. In conclusion, hsa-miR-15b-5p, hsa-miR-184, and hsa-miR-16-5p are identified as specific related exosomal miRNAs for IS patients. These genes may provide new targets for the early identification of IS.

## 1. Introduction

Ischemic stroke (IS) is one of the leading causes of disability and mortality worldwide and accounts for 80% of all strokes [1]. Intravenous thrombolysis is the only approved treatment strategy for acute IS by FDA [2]. Due to the short treatment time window within 4.5 h of onset and the high risk of hemorrhage, the thrombolysis rate in IS patients is very low [3], suggesting early diagnosis is extremely critical. Nowadays, clinical diagnosis of IS mainly relies on magnetic resonance imaging and computed tomography [4]. However, most community medical institutions lack the testing equipment, and the brain imaging examinations are relatively expensive, which limits the clinical diagnosis of IS [5]. Thus, developing new diagnostic markers and therapeutic targets for IS are urgently needed.

microRNAs (miRNAs) are a class of small and noncoding RNA, which regulate gene expression through silencing target genes and play important roles in the nervous system diseases, such as traumatic brain injury, spinal cord injury, subarachnoid hemorrhage, and IS [6–8]. For example, miR-31 inhibits traumatic brain injury-triggered neuronal cell apoptosis by regulating hypoxia-inducible factor-1A/vascular endothelial growth factor A axis [7]. miR-672-3p promotes functional recovery in rats with contusive spinal cord injury by inhibiting ferroptosis suppressor protein 1 [8]. Nowadays, more and more studies identify that some aberrant expressed circulating miRNAs in the serum, which are identified via bioinformatics methods, could be used as potential candidates for disease diagnosis. For example, Hsa-miR-484, hsa-miR-185-5p, hsa-miR-340-5p, hsa-miR-146a-5p, and hsa-miR-195-5p have a prognostic value in breast cancer patients treated with integrative interventions, including diet and physical activity [9]. Specific alteration of 20 miRNAs has a direct association with pesticide exposure and the development of neurodegenerative diseases [10]. Hsa-miR-181a-3p, hsa-miR-214-3p, hsa-miR-18a-5p, and hsa-miR-938 are positively related to the IL-6 signaling pathway activation in various cancers [11]. 14 potentially important miRNAs are identified as IS diagnostic signatures via bioinformatics method combined with logistic regression analysis [12]. Whereas circulating miRNAs in the serum are generally affected by the different pathophysiological conditions and circulating ribonucleases [13], there are still short of reliable prognostic or diagnostic blood biomarkers.

Exosomes are spherical extracellular nanovesicles with a bilayer lipid structure for the outer membrane, defined by a diameter of 30–100 nm [14]. Exosomes contain proteins, mRNAs, and miRNAs and play essential roles in intercellular communication [15]. Due to their high stability, serum exosomal miRNAs are considered to be powerful non-invasive biomarkers in many diseases, including breast cancer, pancreatic ductal adenocarcinoma, and hepatocellular carcinoma [16–18]. Recently, Tong et al. [19] found that miR-151a-5p, miR-24, mir-485-5p, mir-331-5p, and mir-214 were upregulated with statistical significance in both the serum exosome and cerebrospinal fluid exosomes, and may be considered as a biomarker for the diagnosis of Parkinson's disease.

In this study, we attempt to identify the crucial exosomal miRNAs associated with IS by using bioinformatics methods, reveal potential biomarkers for IS, and investigate the association between the identified biomarker and immune cell infiltration in IS.

## 2. Materials and Methods

### 2.1. Data Sources

The data were downloaded from the Gene Expression Omnibus (GEO) database (http://www.ncbi.nlm.nih.gov/geo). The inclusion and exclusion criteria for these data sets were as follows. Inclusion criteria: (i) data sets containing miRNA and mRNA expression levels of IS patients; (ii) data sets reporting miRNAs expression levels of both IS patient and healthy patient; and (iii) data sets containing miRNA expression data of at least 5 IS samples and 5 controls. Exclusion criteria: (i) data sets containing exclusively IS sample and (ii) data sets containing miRNAs expression levels of animal models, cell lines or other in vitro experiments.

GSE199942 included the miRNA expression profiles of serum exosomes in five acute IS patients and five healthy controls detected by RNA-seq using llumina HiSeqTM 2500. GSE22255 included the gene expression profiling in peripheral blood mononuclear cells (PBMCs) of 20 IS patients and 20 sex- and age-matched controls using Affymetrix microarrays. GSE16561 included the gene expression profiles of peripheral whole blood RNA from 39 IS patients and 24 healthy controls. GSE117064 included the microRNA profiles of 1785 samples, which consist of 173 of CVD patients, 1612 of non-CVD control using microarrays. PMC8702168 included the miRNA expression profiles of human stroke brain tissue in five acute IS patients and three non-stroke controls detected by RNA-seq using Illumina NextSeq 500. All the data sets used in this study could be available on GEO data sets.

### 2.2. Analysis of Differentially Expressed miRNAs and Genes

The differentially expressed miRNAs from GSE199942 were obtained using the edgeR package [20]. The differentially expressed genes from GSE22255 were obtained using the limma package [21]. The *P* value <0.05 was used as threshold for nominally significant differential expression. gplot2 and pheatmap packages were used to draw the volcanic maps and heat maps, respectively.

### 2.3. Sankey Diagram of the miRNAs-mRNAs Network

The miRNAs-mRNAs network was drawn using the “ggalluvial” package of R software.

### 2.4. GO and KEGG Analysis

Gene ontology (GO) functional enrichment and Kyoto Encyclopedia of Genes and Genomes (KEGG) pathway were performed using the “clusterProfiler” R package [22]. *P* value <0.05 was considered the criterion for statistical significance.

### 2.5. Evaluation of Immune Cell Abundance

The gene expression profiling of GSE22255 was used to compare immune cell abundance in the peripheral blood of 20 IS patients and 20 healthy controls. The “CIBERSORT” algorithm was used to calculate the relative proportions of 22 types of immune cells [23]. Significant alterations in immune cells were identified using the Wilcoxon test at *P* < 0.05. The “ggcorrplot” package was used to visualize the results of correlation between immune cells.

### 2.6. Diagnostic Value of Differentially Expressed Genes in IS

The receiver operating characteristic (ROC) curve analysis is often used to determine the best diagnostic threshold for a diagnostic method. Generally, the area under the curve (AUC) value of >0.7 obtained from ROC curve analysis is considered to have a good predictive value [24].

## 3. Results

### 3.1. Identification of Differentially Expressed Exosomal miRNAs Related to IS

**Figure 1:**
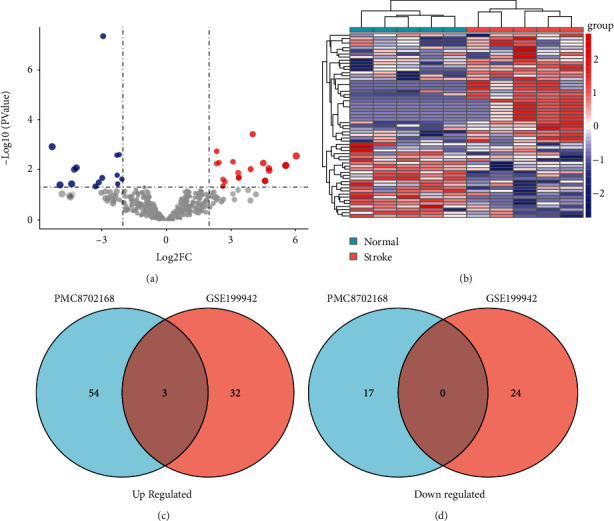
Scanning differentially expressed miRNAs. (a) Differentially expressed miRNAs in GSE199942. |LogFC| >1 and *P* value <0.05 were set to screen. (b) Heatmap of the differentially expressed miRNAs in GSE199942. (c) Venn diagram representing the intersection of up-regulated expressed miRNAs in the 2 data sets (GSE199942 and PMC8702168). (d) Venn diagram representing the intersection of down-regulated expressed miRNAs in the 2 data sets (GSE199942 and PMC8702168).

In this study, we analyzed the differentially expressed miRNAs in serum *exosomes* between IS patients and healthy controls from GSE199942 data sets, and found that 35 upregulated and 24 downregulated miRNAs (Table 1). The expression of these miRNAs is shown in Figures 1(a) and 1(b). Furthermore, the differentially expressed serum *exosomes* miRNAs were intersected with those differentially expressed miRNAs from freshly removed human stroke brain tissue [25], and three upregulated miRNAs were obtained: hsa-miR-15b-5p, hsa-miR-184, and hsa-miR-16-5p (Figures 1(c) and 1(d)).

### 3.2. Construction of miRNA-mRNA Network

**Figure 2:**
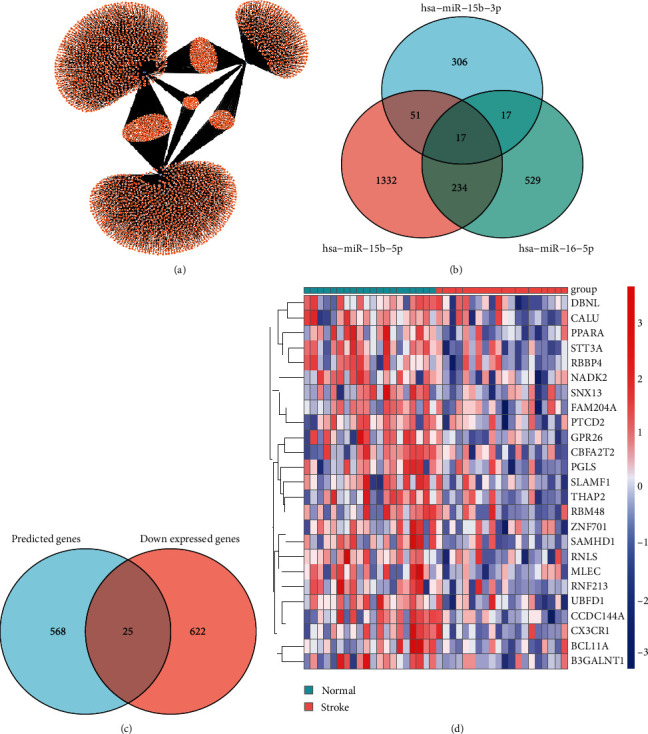
Construction of miRNA-mRNA Network. (a) The miRNA-target gene network for differentially expressed miRNAs. (b) Venn diagram showing the overlap among the predicted targets of the differentially expressed miRNAs. (c) Venn diagram showing the overlap between down-regulated expressed genes and predicted targets of the differentially expressed miRNAs. (d) Heat map of the 25 intersected genes.

**Figure 3:**
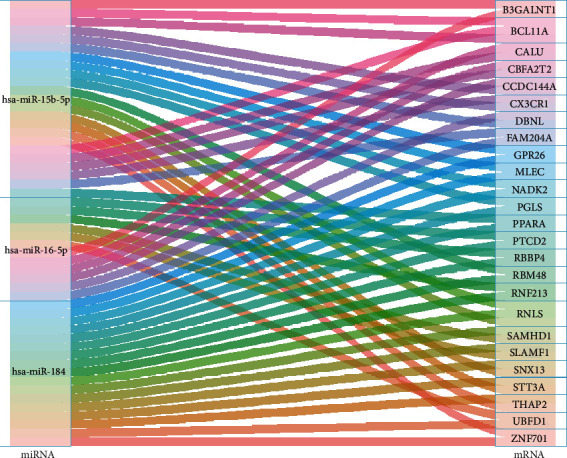
Sankey diagram of the miRNA-mRNA Network in IS.

Next, we analyzed the potential targets of the three differentially expressed serum microvesicles miRNAs by miRWalk, and found a total of 593 genes may be co-regulated by at least two of the three miRNAs, of which 192 genes were co-targeted by hsa-miR-15b-5p and hsa-miR-16-5p, 250 genes were co-targeted by hsa-miR-15b-5p and hsa-miR-184, 92 genes were co-targeted by hsa-miR-16-5p and hsa-miR-184, and 59 genes were co-targeted by the three miRNAs (Figures 2(a) and 2(b) and Table 2). Given that miRNA negatively regulates the expressions of its targets, we further intersected the 593 prediction genes with the downregulated-expressed genes of PBMCs of IS patients from GSE22255 [26], and obtained 25 genes (Figure 2(c)). The expression of the 25 intersected genes was shown in Figure 2(d). Then, the network containing 3 miRNAs and 25 mRNAs was visualized by the “ggallouvial” package of R software and was shown in Figure 3.

### 3.3. Enrichment Analysis of Differentially Expressed Genes

**Figure 4:**
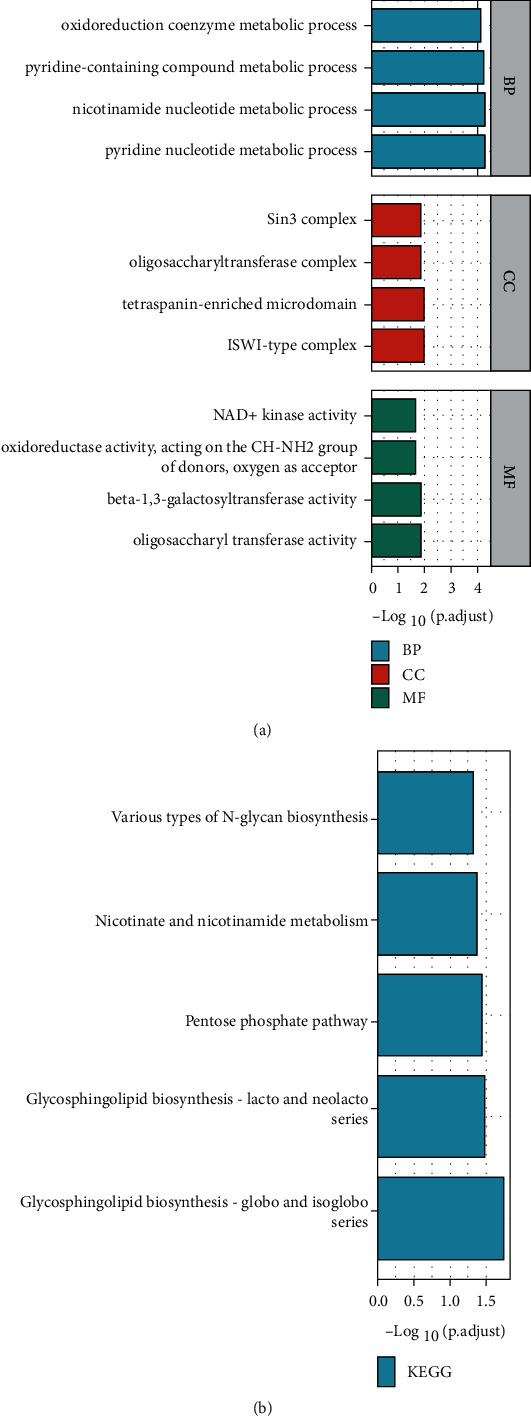
GO (a) and KEGG (b) analysis of the 25 intersected genes.

To explore the potential biological functions of the above downregulated-expressed 25 mRNAs, we performed GO annotation and KEGG pathway enrichment analysis. The enriched GO annotation included oxidoreduction coenzyme metabolic process, pyridine-containing compound metabolic process, and nicotinamide nucleotide metabolic process in the biological process (BP) category. Sin3 complex, oligosaccharyltransferase complex, and tetraspanin-enriched microdomain were included in the cellular component (CC) category. NAD+ kinase activity, oxidoreductase activity, and beta-1,3-galactosyltransferase activity were included in the molecular function (MF) category (Figure 4(a)). KEGG pathway analysis showed these genes were mainly involved in various types of N-glycan biosynthesis, nicotinate and nicotinamide metabolism, and pentose phosphate pathway (Figure 4(b)).

### 3.4. PPI Network Construction and Validation of Predictive Feature Biomarkers

**Figure 5:**
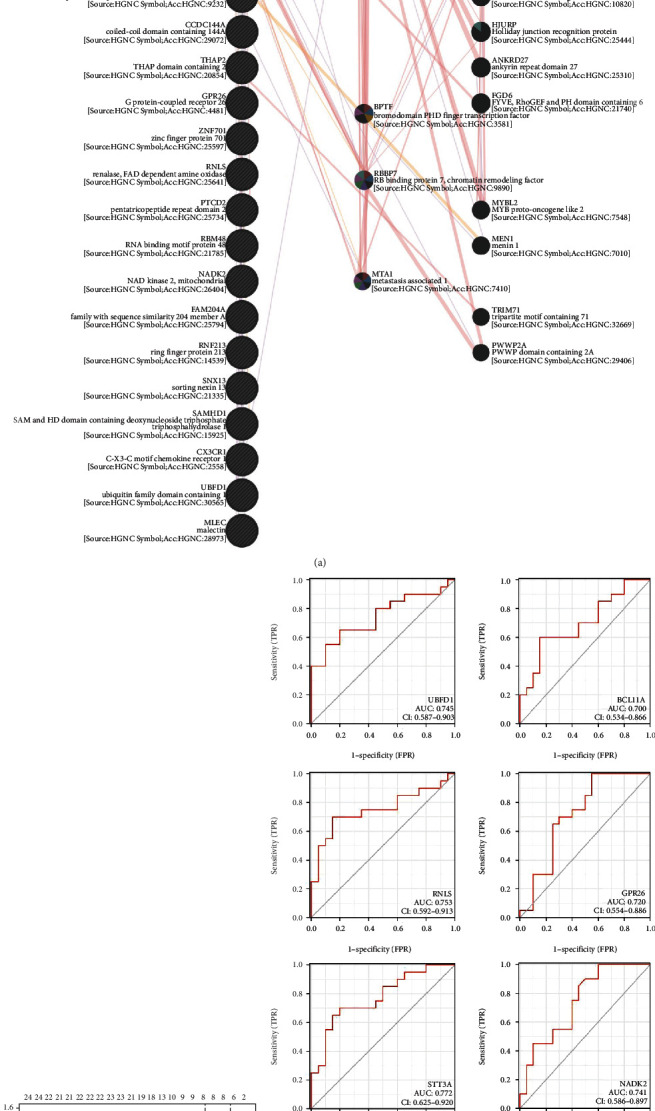
PPI network construction and validation of predictive feature biomarkers. (a) PPI network of the 25 intersected genes constructed by GENEMANIA. (b) A plot of biomarkers selection by LASSO regression algorithm. (c) ROC diagnostic curve for the eight genes selected by ASSO regression algorithm.

We further analyzed the protein-protein interaction (PPI) network of the above 25 intersected genes by Genemania and identified the RB binding protein 4 (RBBP4) had the most interactions in the network (Figure 5(a)). Furthermore, we performed the LASSO algorithm to narrow down the above 25 intersected genes and identified eight key genes related to IS (Figure 5(b)). The eight genes were BCL11A, RNLS, UBFD1, STT3A, NADK2, GPR26, PPARA, and SAMHD1. Moreover, we used ROC curve to estimate the predictive value of the eight genes, and found that all of the eight genes had a certain diagnostic value in discriminating IS patients from the healthy controls, with an AUC of 0.7 (Figure 5(C)).

### 3.5. Immune Cell Patterns in the Peripheral Bloods of IS

**Figure 6:**
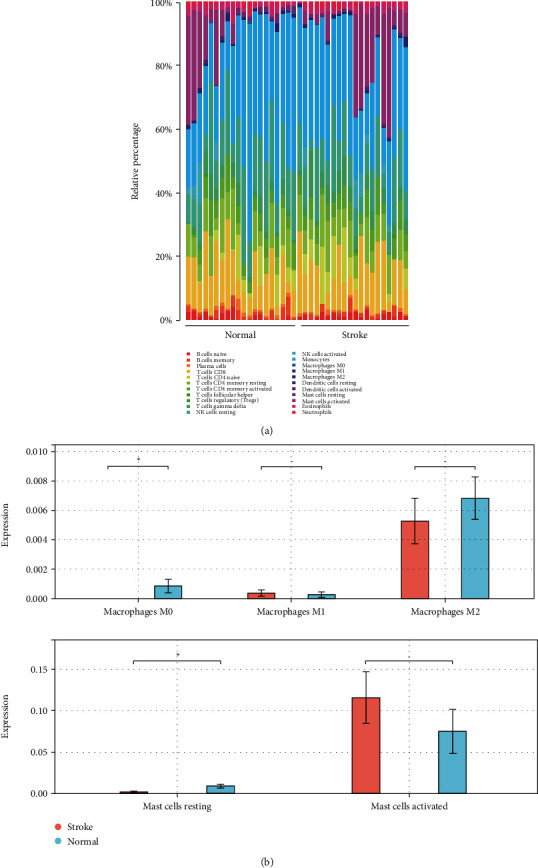
Immune cell patterns in the peripheral bloods of IS samples. (a) The composition of immune cells in the peripheral bloods of IS patients. (b) Comparison of the indicated immune cell subtypes between stroke and normal control group. ^∗^*P* < 0.05.

**Figure 7:**
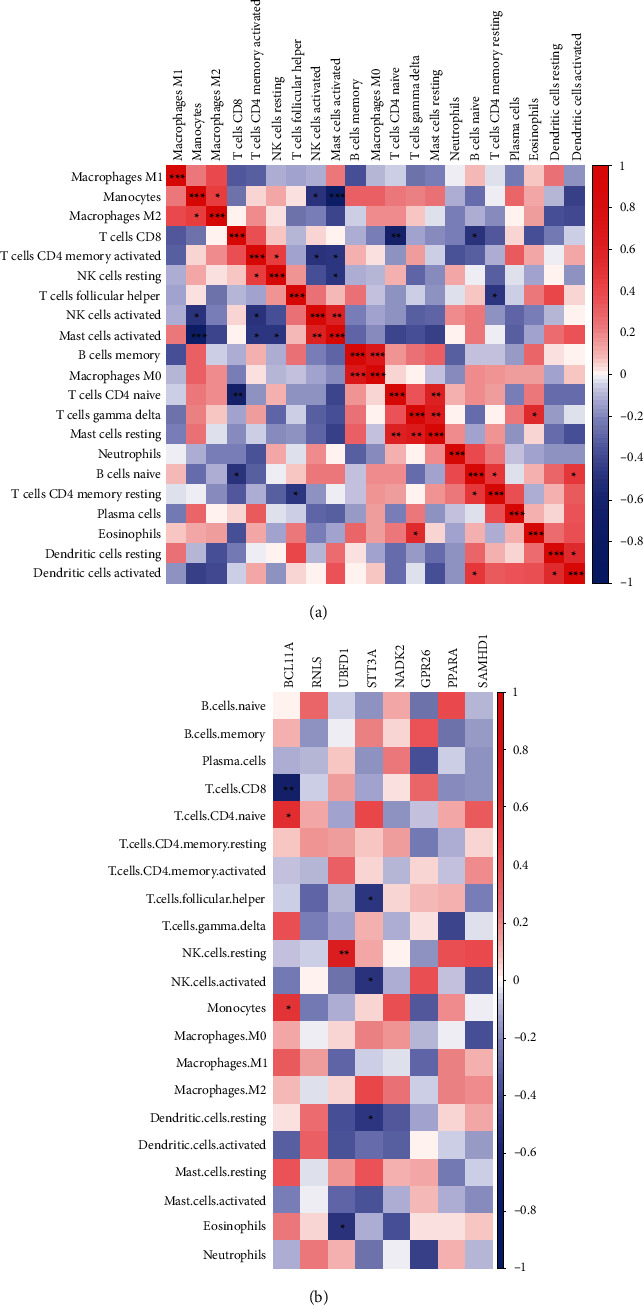
Correlation analysis of immune cells in the peripheral bloods of IS. (a) The correlation between immune cells in IS samples. (b) The correlation between the eight key genes and immune cells. ∗*P* < 0.05, ∗∗*P* < 0.01, ∗∗∗*P* < 0.001.

Given that peripheral immune system has been shown to play crucial roles in the evolution of ischemic brain damage [27, 28], we further compared the different immune cell patterns of the peripheral bloods between IS patients and normal controls from GSE22255. As shown in Figures 6(a) and 6(b), M0 macrophages and resting mast cells were significantly decreased in the peripheral bloods of IS patients. M1 macrophages and activated mast cells were slightly increased in the IS group but had no significant difference compared to the normal control group (Figure 6(b)). Furthermore, we analyzed the correlation among the immune cells and found that monocytes were significantly positively correlated to M2 macrophages and significantly negatively correlated to activated NK cells and activated mast cells (Figure 7(a)). Moreover, we analyzed the correlation between the immune cells and the eight variables related to IS and found that STT3A was significantly negative-correlated to follicular helper T cells, activated NK cells and resting dendritic cells (Figure 7(b)).

### 3.6. Verification of the Diagnostic Efficacy of the Key miRNAs

**Figure 8:**
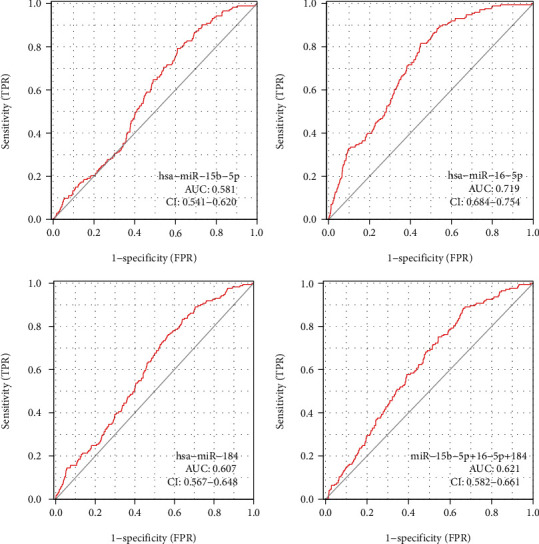
Verification of the diagnostic efficacy of the key miRNAs. ROC diagnostic curve for the three miRNAs.

GSE117064 were included in this study as the verification series to validate the diagnostic efficacy of the above three miRNAs. Has-miR-16-5p was more advantageous for diagnosing stroke than the other two miRNAs, as shown in Figure 8.

## 4. Discussion

Stroke causes great burden on human health and the economy. Although prominent improvements in the early diagnosis and treatment of IS in the past decade, it is still a leading cause of death and disability. Thus, identifying new biomarkers for the early diagnosis of IS is essential. Recently, serum glutamic oxaloacetic transaminase is reported to may be utilized as predictor in detection of early neurological deterioration in acute IS [29]. Here, we first used the GEO data set to detect the differentially expressed exosomal miRNAs associated with IS and identified three miRNAs (hsa-miR-15b-5p, hsa-miR-184, and hsa-miR-16-5p) intersected with those differentially expressed miRNAs from freshly removed human stroke brain tissue [25]. Among the predicted target genes of the three differentially expressed exosomal miRNAs, 25 mRNAs were obtained by intersecting with differentially expressed genes from GSE22255. Then, we used LASSO algorithm to identify eight key genes related to IS, and further analyzed the independent prediction ROC curve. Subsequently, we analyzed the immune cell patterns in the peripheral bloods of IS patients and the correlation between the immune cells and the eight key genes as potential biomarkers for IS.

Previous studies have reported the significance of the miRNAs, which were identified in our study. hsa-miR-15b-5p is highly expressed in the plasma of Alzheimer's disease (AD) patients, and could distinguish AD patients from normal controls [30, 31]. Besides, hsa-miR-15b-5p in the aqueous humour of patients with diabetic macular oedema shows a fold change greater than –50 in log2 values, compared to the control group [32]. In this study, we firstly identified hsa-miR-15b-5p is highly expressed in the exosomes of IS patients. Recently, hsa-miR-15b-5p is found to regulate the proliferation and apoptosis of human vascular smooth muscle cells [33]. However, the specific mechanism of action of hsa-miR-15b-5p in stroke is unclear, and needs further investigation. miR-16-5p is found to be highly expressed in the in serum and plasma of patients with colorectal cancer [34], and in the feces of patients with precancerous lesions [35]. Besides, hsa-miR-16-5p may be a good candidate for identification of predictive biomarkers of duloxetine response in patients with major depressive disorder who are responsive to duloxetine treatment [36], suggesting that hsa-miR-16-5p may play important roles in the nervous system disease. Consistently, we also found hsa-miR-16-5p is highly expressed in the serum of IS patients, and the specific role of hsa-miR-16-5p in IS needs further investigation. Hsa-miR-184 has been proposed as a biomarker for several cancers, including non-small cell lung, uterine corpus endometrial carcinoma, and oral squamous cell carcinoma [37–39], and brain-enriched hsa-miR-184 is downregulated in older adults with major depressive disorder [40]. Here, we identified that hsa-miR-184 is highly expressed in the serum of IS patients. Moreover, the co-regulated genes of the above three miRNAs were enriched in the N-glycan biosynthesis, nicotinate and nicotinamide metabolism, and pentose phosphate pathways. Interestingly, N-glycosylated immunoglobulin G has been shown to be associated with IS [41], and plasma N-glycans could as emerging biomarkers of cardiometabolic risk [42]. How the three miRNAs regulating N-glycan biosynthesis to affect the IS development would be explored in our following studies, and it could be a very interesting area.

Peripheral immune cells interplay with the central nervous system, and play important roles in the brain development, and in the process of IS injury [43, 44]. The immune system is rapidly activated after IS, and peripheral immune cells migrate and infiltrate across the blood–brain barrier into the ischemic region to affect infarction progression and prognosis. Leukocytes are found to aggregate in the ischemic region of MCAO models as early as 30 min after occlusion [45]. Yilmaz et al. fond that CD4+ and CD8+ T cells are distributed in the brain tissue 24 h after IS onset, and act detrimental effects on post-ischemic cerebral immune responses [46]. In this study, we analyzed the different immune cell patterns of the peripheral bloods between IS patients and normal controls from GSE22255, and found M0 macrophages and resting mast cells were significantly decreased in the IS group, and pro-inflammatory M1 macrophages, and activated mast cells were slight increased, suggesting that the inflammatory responses were rapidly activated after stroke. Our result is consistent with recent study that CD11b + CD45+ and CD11b + Ly6G- monocytes and macrophages were greatly increased 3 days after cardiac arrest and resuscitation [47]. Mast cells are rapidly activated and release TNF-*α* and histamine to aggravate brain damage after IS [48, 49]. Among the eight key genes potentially targeted by the above three miRNAs identified in our study, STT3A, a catalytic subunit of the oligosaccharyltransferase, increases amyloid-*β* production by promoting N-glycosylation in the pathogenesis of Alzheimer's disease [50]. Besides, the expression level of STT3A was found to be negatively related to the biomarker expressions of M1 macrophages in breast cancer [51], indicating that STT3A may be involved in the regulating macrophage activation. Although the mechanism of regulating macrophages or mast cells has been extensively study, whether hsa-miR-15b-5p/hsa-miR-184/hsa-miR-16-5p-STT3A axis is involved in the regulating macrophages or mast activation in IS need further investigation.

This study has some limitations. For example, our analytical data are derived from public database with relatively small sample sizes. Second, important analysis results need to be further validated with clinical samples. Further large-scale basic studies can be carried out to verify the conclusions of this study.

## 5. Conclusions

We for the first time conducted a comprehensive bioinformatics analysis and identified three serum exosomal miRNAs (hsa-miR-15b-5p, hsa-miR-184, and hsa-miR-16-5p), which may be involved in affecting the peripheral immune cell patterns of IS patients and might serve as promising diagnostic biomarkers. However, the specific pathogenesis and molecular targets still need to be further confirmed through molecular experiments.

## Figures and Tables

**Table 1:** The differentially expressed miRNAs in serum exosomes between IS patients and healthy controls from GSE199942 data sets.

Genes	logFC	logCPM	*P* Value	FDR	Change
hsa-miR-4511	4.0177751	3.1519311	0.000391	0.1276672	Up
hsa-miR-450a-5p	2.3608531	4.75	0.0018718	0.2345272	Up
hsa-miR-15b-5p	1.8701565	5.6824497	0.0027574	0.2345272	Up
hsa-miR-187-3p	6.0409799	3.6370248	0.0028732	0.2345272	Up
hsa-miR-4488	3.107022	5.1401173	0.0049058	0.2367778	Up
hsa-miR-30a-5p	2.4603292	10.202389	0.0051825	0.2367778	Up
hsa-miR-3688-3p	4.5127781	1.9219509	0.0054893	0.2367778	Up
hsa-miR-30a-3p	2.3492478	7.3137497	0.0057183	0.2367778	Up
hsa-miR-519b-5p	5.5515639	2.2856343	0.0067765	0.2367778	Up
hsa-miR-523-5p	5.5515635	2.2856343	0.0067769	0.2367778	Up
hsa-miR-522-5p	5.5515236	2.2856343	0.0068149	0.2367778	Up
hsa-miR-518e-5p	5.55147	2.2856343	0.0068664	0.2367778	Up
hsa-miR-519c-5p	5.5514537	2.2856343	0.0068822	0.2367778	Up
hsa-miR-519a-5p	5.5513848	2.2856343	0.0069491	0.2367778	Up
hsa-miR-23a-3p	1.3900129	8.9137221	0.0073152	0.2367778	Up
hsa-miR-223-3p	1.6990938	6.5924347	0.0076146	0.2367778	Up
hsa-miR-1262	4.7679363	1.8418462	0.0090333	0.2564662	Up
hsa-miR-184	3.9283866	3.4873381	0.0096432	0.2623126	Up
hsa-let-7g-3p	4.777119	1.8349666	0.010846	0.2623126	Up
hsa-miR-503-5p	1.4637255	5.9719843	0.011694	0.2727198	Up
hsa-miR-2115-3p	3.3598963	2.5755635	0.0141269	0.3180986	Up
hsa-miR-365a-3p	3.3751344	3.1958665	0.0210196	0.4111132	Up
hsa-miR-365b-3p	3.3752176	3.1958665	0.0213312	0.4111132	Up
hsa-miR-143-3p	1.3106202	12.340244	0.0232167	0.4123441	Up
hsa-miR-140-3p	1.1006504	13.62317	0.0239955	0.4123441	Up
hsa-miR-4677-3p	2.6511654	2.7753844	0.0247074	0.4136901	Up
hsa-miR-708-3p	1.5837876	4.899895	0.0268826	0.4341984	Up
hsa-miR-518d-5p	4.5933127	1.7706809	0.0284491	0.4341984	Up
hsa-miR-520c-5p	4.5932967	1.7706809	0.0284886	0.4341984	Up
hsa-miR-526a	4.5932549	1.7706809	0.0285919	0.4341984	Up
hsa-miR-16-5p	1.1811937	10.014053	0.0295433	0.4384491	Up
hsa-miR-873-5p	2.7302829	3.6544356	0.0305988	0.4440228	Up
hsa-miR-218-5p	1.673055	4.8719318	0.0327228	0.4546377	Up
hsa-miR-542-3p	1.702724	4.0029647	0.0387468	0.4914672	Up
hsa-miR-501-5p	2.6408656	1.9304482	0.0466	0.5261696	Up
hsa-miR-185-5p	–0.995934	14.161013	0.0511742	0.5569459	No significant change
hsa-miR-206	–1.392741	5.7462987	0.0633447	0.6573682	No significant change
hsa-miR-7113-5p	3.8099703	1.6612046	0.0652589	0.6573682	No significant change
hsa-miR-509-3-5p	3.3464599	2.1064268	0.0670369	0.6573682	No significant change
hsa-miR-548ad-5p	1.5915279	4.2304639	0.0679466	0.6573682	No significant change
hsa-miR-548ae-5p	1.5915235	4.2304639	0.0684497	0.6573682	No significant change
hsa-miR-107	0.9647205	7.8566926	0.069841	0.6573682	No significant change
hsa-miR-6511b-5p	–1.909183	3.2621364	0.0716849	0.6573682	No significant change
hsa-miR-6741-3p	–2.264989	2.2929563	0.0723131	0.6573682	No significant change
hsa-miR-145-3p	1.0398494	6.7240529	0.0723487	0.6573682	No significant change
hsa-miR-204-3p	1.6478542	5.208978	0.0728423	0.6573682	No significant change
hsa-miR-330-3p	–0.869386	7.1790501	0.0731215	0.6573682	No significant change
hsa-miR-4762-3p	–2.799118	2.0076936	0.0754312	0.6573682	No significant change
hsa-miR-589-5p	0.8636502	6.1429256	0.0773701	0.6573682	No significant change
hsa-miR-671-3p	–1.041089	5.1409541	0.0790674	0.6573682	No significant change
hsa-miR-323b-3p	2.2265637	3.0213546	0.0792916	0.6573682	No significant change
hsa-miR-125a-5p	–1.112356	7.7678399	0.0798332	0.6573682	No significant change
hsa-miR-141-3p	–2.577872	2.3667262	0.0800967	0.6573682	No significant change
hsa-miR-3913-5p	1.0922674	4.8446192	0.0813834	0.6573682	No significant change
hsa-miR-378c	–1.333366	9.2448839	0.0814884	0.6573682	No significant change
hsa-miR-744-5p	–0.797828	9.5563371	0.0822931	0.6573682	No significant change
hsa-miR-148a-5p	–1.102375	5.6535438	0.0842296	0.6573682	No significant change
hsa-miR-5193	–2.996555	1.7341056	0.084313	0.6573682	No significant change
hsa-miR-1224-5p	–1.333808	5.0287236	0.0856865	0.6573682	No significant change
hsa-miR-1298-5p	2.1271815	2.4541849	0.0863259	0.6573682	No significant change
hsa-miR-548ay-5p	1.480535	3.4698845	0.0885952	0.6573682	No significant change
hsa-miR-326	–1.490204	3.2492	0.0888616	0.6573682	No significant change
hsa-miR-4755-5p	–1.963428	2.9818739	0.0889711	0.6573682	No significant change
hsa-miR-331-5p	1.3601536	3.3388849	0.0891202	0.6573682	No significant change
hsa-miR-27a-5p	0.9183589	6.5858081	0.0914764	0.6573682	No significant change
hsa-miR-3928-3p	–1.271881	5.1073389	0.0930995	0.6573682	No significant change
hsa-miR-509-3p	3.1073088	2.4748986	0.0935276	0.6573682	No significant change
hsa-miR-3157-3p	1.6474404	2.5354758	0.0939205	0.6573682	No significant change
hsa-miR-6810-5p	–4.812779	1.8099467	0.0941476	0.6573682	No significant change
hsa-miR-27a-3p	1.0144034	9.7833195	0.0946288	0.6573682	No significant change
hsa-miR-15b-3p	1.8701193	3.380578	0.0968837	0.6590269	No significant change
hsa-miR-34a-5p	4.1686458	1.6079117	0.0977092	0.6590269	No significant change
hsa-miR-3130-3p	1.5038518	3.6004664	0.099497	0.6590269	No significant change
hsa-miR-371a-5p	–4.337354	1.6313631	0.0995531	0.6590269	No significant change
hsa-miR-1292-5p	–1.033145	5.8012275	0.1007279	0.6590269	No significant change
hsa-miR-214-3p	1.6997602	2.9803798	0.1017758	0.6590269	No significant change
hsa-miR-1307-3p	–0.862513	10.95775	0.1019322	0.6590269	No significant change
hsa-miR-1246	1.0685772	11.120133	0.1060379	0.6612336	No significant change
hsa-miR-6747-3p	–2.403327	1.9904787	0.1087132	0.6612336	No significant change
hsa-miR-6857-3p	–2.313114	1.8454211	0.1093797	0.6612336	No significant change
hsa-miR-6859-5p	–1.293482	3.0140416	0.1099169	0.6612336	No significant change
hsa-miR-2277-3p	–4.486188	1.684403	0.1104925	0.6612336	No significant change
hsa-miR-1273h-3p	–1.024439	4.7744306	0.1125173	0.6612336	No significant change
hsa-miR-4489	–1.652656	2.8115404	0.1157286	0.6612336	No significant change
hsa-miR-2110	–0.763399	9.533566	0.1160144	0.6612336	No significant change
hsa-miR-582-3p	0.9692591	6.8717413	0.1178804	0.6612336	No significant change
hsa-miR-421	1.1333857	3.7603465	0.1179684	0.6612336	No significant change
hsa-miR-6808-3p	–1.864298	2.0341099	0.118289	0.6612336	No significant change
hsa-miR-6855-5p	2.3206825	2.6920197	0.1185141	0.6612336	No significant change
hsa-miR-30d-3p	1.9658383	2.2380137	0.1185774	0.6612336	No significant change
hsa-miR-548g-3p	2.1365963	1.9277346	0.1202941	0.6612336	No significant change
hsa-miR-4632-5p	–4.438452	1.6646651	0.1206023	0.6612336	No significant change
hsa-miR-223-5p	1.0326838	10.994597	0.1223615	0.6612336	No significant change
hsa-miR-148b-3p	0.8571842	9.1175856	0.1230929	0.6612336	No significant change
hsa-miR-511-5p	–0.954287	7.6962227	0.1233912	0.6612336	No significant change
hsa-miR-3120-5p	–1.569549	3.189544	0.1238989	0.6612336	No significant change
hsa-miR-6807-5p	–1.68445	2.7656362	0.1258088	0.6612336	No significant change
hsa-miR-7706	–0.800976	8.1364183	0.1259873	0.6612336	No significant change
hsa-miR-6877-5p	–0.97864	4.0346695	0.1281799	0.6612336	No significant change
hsa-miR-6867-5p	–4.420387	1.6399348	0.128713	0.6612336	No significant change
hsa-miR-654-3p	1.4716482	4.058458	0.1292771	0.6612336	No significant change
hsa-miR-532-3p	1.3345266	3.0579425	0.1310935	0.6612336	No significant change
hsa-miR-139-5p	–0.763961	8.7983053	0.1311084	0.6612336	No significant change
hsa-miR-150-5p	–0.951721	4.5776261	0.1315027	0.6612336	No significant change
hsa-miR-29c-3p	1.590568	2.9374216	0.1321838	0.6612336	No significant change
hsa-miR-216b-5p	2.2373978	2.203677	0.1325936	0.6612336	No significant change
hsa-miR-182-5p	0.9661418	8.8897524	0.1333784	0.6612336	No significant change
hsa-miR-422a	–1.078467	4.6298508	0.1338046	0.6612336	No significant change
hsa-miR-598-5p	–2.666613	1.5735392	0.134677	0.6612336	No significant change
hsa-miR-5001-5p	–2.551553	2.0369642	0.1370473	0.6620332	No significant change
hsa-miR-134-5p	–0.980716	9.4215516	0.1373406	0.6620332	No significant change
hsa-miR-1284	2.4145406	2.1968535	0.1378813	0.6620332	No significant change
hsa-miR-3150a-3p	–2.811773	1.5634735	0.1390433	0.6627395	No significant change
hsa-miR-363-3p	0.7073762	9.1043511	0.1413506	0.6688546	No significant change
hsa-miR-1228-5p	–1.583718	5.6267219	0.1510184	0.6974406	No significant change
hsa-miR-451b	1.5556534	2.341655	0.1513468	0.6974406	No significant change
hsa-miR-4690-3p	–1.585332	2.3122693	0.1546086	0.6974406	No significant change
hsa-miR-4492	1.8890749	2.6908575	0.1556186	0.6974406	No significant change
hsa-miR-22-5p	0.7113484	7.5008618	0.1564473	0.6974406	No significant change
hsa-miR-382-3p	2.6618706	1.8884254	0.1569376	0.6974406	No significant change
hsa-miR-1268a	–1.344074	3.5173016	0.157215	0.6974406	No significant change
hsa-miR-130b-5p	–1.349846	5.5368302	0.1573153	0.6974406	No significant change
hsa-miR-1908-5p	–0.868376	5.3205733	0.1575061	0.6974406	No significant change
hsa-miR-1323	3.1754272	1.7499644	0.1587152	0.6974406	No significant change
hsa-miR-142-5p	0.9245943	10.746901	0.1591403	0.6974406	No significant change
hsa-miR-202-5p	–2.148219	2.1091604	0.1611812	0.7016757	No significant change
hsa-miR-3614-5p	0.8235881	5.10239	0.1628295	0.7029907	No significant change
hsa-miR-4734	–2.388663	2.0593932	0.1636364	0.7029907	No significant change
hsa-miR-500a-3p	0.8190993	6.9227609	0.1671241	0.7088167	No significant change
hsa-miR-6837-3p	2.3338704	1.5337101	0.1671635	0.7088167	No significant change
hsa-miR-1251-5p	3.9176501	1.5341643	0.1714535	0.7155546	No significant change
hsa-miR-1303	–1.555532	2.6559909	0.1716289	0.7155546	No significant change
hsa-miR-6729-5p	–1.590327	2.658534	0.1725684	0.7155546	No significant change
hsa-miR-3164	–1.40814	2.542441	0.1747991	0.7155546	No significant change
hsa-miR-4686	–2.712178	1.7671251	0.1754859	0.7155546	No significant change
hsa-miR-199a-5p	–0.808327	5.143726	0.1766325	0.7155546	No significant change
hsa-let-7i-5p	0.6705203	13.732316	0.1767354	0.7155546	No significant change
hsa-miR-98-5p	1.0003412	5.4352618	0.1775189	0.7155546	No significant change
hsa-miR-148a-3p	0.9435646	16.290335	0.1788868	0.7166447	No significant change
hsa-miR-424-5p	1.8940264	2.2198714	0.1808803	0.7189678	No significant change
hsa-miR-4738-3p	–0.927843	4.9421983	0.1816687	0.7189678	No significant change
hsa-miR-548av-5p	1.8025998	2.5786282	0.1845222	0.7226147	No significant change
hsa-miR-548k	1.8026604	2.5786282	0.1850914	0.7226147	No significant change
hsa-miR-10b-5p	0.9865763	13.490142	0.1869494	0.7226147	No significant change
hsa-miR-6894-5p	–2.424955	1.6233922	0.1874297	0.7226147	No significant change
hsa-miR-502-3p	0.7905524	6.7211564	0.1881233	0.7226147	No significant change
hsa-miR-548d-5p	1.243827	2.8600041	0.1906509	0.728041	No significant change
hsa-miR-197-5p	2.5681216	1.5569566	0.193898	0.7361359	No significant change
hsa-miR-3177-3p	–0.827688	4.3828417	0.2048054	0.7701355	No significant change
hsa-miR-483-3p	–1.974456	2.2211816	0.2052122	0.7701355	No significant change
hsa-miR-4443	1.441188	2.0131336	0.209035	0.7769258	No significant change
hsa-miR-450b-5p	1.2773184	3.6255182	0.2094011	0.7769258	No significant change
hsa-miR-3944-5p	–1.917286	1.6616247	0.2124654	0.7809496	No significant change
hsa-miR-378a-3p	–0.693553	13.529209	0.2134582	0.7809496	No significant change
hsa-miR-1268b	–1.253701	2.835488	0.2140735	0.7809496	No significant change
hsa-miR-1301-3p	–0.672448	6.3965039	0.2153159	0.7811183	No significant change
hsa-miR-23b-3p	0.8317205	6.7190126	0.2166784	0.7817181	No significant change
hsa-let-7f-5p	0.9813044	12.771715	0.2203357	0.7827953	No significant change
hsa-miR-323a-3p	1.5421509	2.933777	0.2206817	0.7827953	No significant change
hsa-miR-146a-3p	–1.471724	2.8336003	0.2229831	0.7827953	No significant change
hsa-miR-3615	–0.560017	11.59087	0.2231573	0.7827953	No significant change
hsa-miR-3653-3p	1.7884659	2.8943771	0.22665	0.7827953	No significant change
hsa-miR-4750-5p	2.1988462	2.2882648	0.2270104	0.7827953	No significant change
hsa-miR-30b-5p	–1.173984	2.8123367	0.2281171	0.7827953	No significant change
hsa-miR-4685-3p	1.7002163	2.3598704	0.2284197	0.7827953	No significant change
hsa-miR-29a-3p	0.6296572	7.1061101	0.2285709	0.7827953	No significant change
hsa-miR-338-5p	0.9831061	5.1541294	0.2289646	0.7827953	No significant change
hsa-miR-409-5p	–2.455029	1.8660302	0.2303949	0.7835825	No significant change
hsa-miR-6735-5p	–1.113351	2.7252862	0.2358695	0.7980454	No significant change
hsa-miR-451a	0.692269	17.308079	0.2385771	0.8030457	No significant change
hsa-miR-543	–0.786827	5.8479538	0.2440816	0.8039067	No significant change
hsa-miR-320a	–0.815342	17.450679	0.244879	0.8039067	No significant change
hsa-miR-9-5p	–1.371276	3.587857	0.2459402	0.8039067	No significant change
hsa-miR-4669	–1.595144	5.8693776	0.2463495	0.8039067	No significant change
hsa-miR-423-5p	0.9427992	14.818582	0.247431	0.8039067	No significant change
hsa-miR-505-5p	–1.271211	3.1783629	0.2483359	0.8039067	No significant change
hsa-miR-433-3p	–0.847541	3.9645598	0.2494875	0.8039067	No significant change
hsa-miR-378f	–0.711048	5.4020575	0.2506752	0.8039067	No significant change
hsa-miR-369-5p	1.4864754	2.4692721	0.2510632	0.8039067	No significant change
hsa-miR-2355-3p	–1.15072	3.7739493	0.2511439	0.8039067	No significant change
hsa-miR-203a-3p	–1.009401	2.9550279	0.2524344	0.8040958	No significant change
hsa-miR-324-5p	–1.159804	2.7822215	0.2539581	0.8050225	No significant change
hsa-miR-6866-5p	1.21584	2.6771394	0.2586937	0.8156615	No significant change
hsa-miR-146b-5p	–0.50629	10.058382	0.2598126	0.8156615	No significant change
hsa-miR-6850-5p	–1.300932	3.5813908	0.2621724	0.8191319	No significant change
hsa-miR-6805-5p	1.685567	1.9214473	0.2660718	0.8253837	No significant change
hsa-miR-1288-3p	0.7195433	3.7822121	0.2698133	0.8253837	No significant change
hsa-miR-671-5p	–1.32528	2.7635774	0.2698485	0.8253837	No significant change
hsa-miR-3198	1.1473064	2.8345948	0.2707223	0.8253837	No significant change
hsa-let-7g-5p	0.6845773	11.375567	0.2732324	0.8253837	No significant change
hsa-miR-15a-5p	1.1948987	3.6848885	0.2740772	0.8253837	No significant change
hsa-miR-125b-5p	–0.732038	7.8426642	0.2741847	0.8253837	No significant change
hsa-miR-1304-3p	0.9043526	2.9166587	0.2780766	0.8253837	No significant change
hsa-miR-1307-5p	0.9063819	5.2243053	0.2796302	0.8253837	No significant change
hsa-miR-1304-5p	0.7762674	4.5305669	0.2802373	0.8253837	No significant change
hsa-let-7a-3p	0.9330917	4.8154346	0.2830936	0.8253837	No significant change
hsa-let-7c-5p	0.6875779	8.8167555	0.2861486	0.8253837	No significant change
hsa-miR-501-3p	0.5004423	8.0575092	0.2867648	0.8253837	No significant change
hsa-miR-4504	1.0350608	2.5326026	0.2872558	0.8253837	No significant change
hsa-miR-6740-5p	–1.581123	1.7343998	0.2885572	0.8253837	No significant change
hsa-miR-548ah-3p	–1.304342	3.3197506	0.2890754	0.8253837	No significant change
hsa-miR-548ah-5p	1.9294896	1.7038912	0.2893556	0.8253837	No significant change
hsa-miR-483-5p	–0.930561	7.564909	0.2914031	0.8253837	No significant change
hsa-miR-660-5p	0.7075408	4.2882236	0.2914257	0.8253837	No significant change
hsa-miR-21-3p	0.9542321	2.4157215	0.2931474	0.8253837	No significant change
hsa-miR-3934-5p	–1.212719	2.7002545	0.2933218	0.8253837	No significant change
hsa-miR-636	–0.964891	3.8563112	0.2935647	0.8253837	No significant change
hsa-miR-548o-3p	0.681933	6.0771291	0.293988	0.8253837	No significant change
hsa-miR-6847-5p	–1.849893	1.800303	0.294509	0.8253837	No significant change
hsa-miR-548e-3p	1.6874698	2.8139597	0.2958437	0.825581	No significant change
hsa-miR-548aq-3p	1.0658174	2.5363606	0.2988553	0.8304362	No significant change
hsa-miR-548p	–1.282776	2.5748882	0.3025163	0.8347429	No significant change
hsa-miR-224-5p	–0.815762	5.4259038	0.3029618	0.8347429	No significant change
hsa-miR-6842-3p	–0.54062	6.4181974	0.30662	0.8353266	No significant change
hsa-miR-6842-5p	–1.10107	3.1350216	0.3067038	0.8353266	No significant change
hsa-miR-664a-5p	–0.511649	7.5691025	0.3070113	0.8353266	No significant change
hsa-miR-29b-3p	0.9135712	3.3857084	0.3104169	0.8410881	No significant change
hsa-miR-4792	1.6281913	1.7670514	0.3132049	0.8451357	No significant change
hsa-miR-374b-5p	1.1494207	2.7976908	0.3161745	0.8482237	No significant change
hsa-miR-548ar-3p	2.0368642	1.7291385	0.3173662	0.8482237	No significant change
hsa-miR-101-3p	0.5091777	12.22281	0.3190473	0.8482237	No significant change
hsa-miR-19b-3p	–0.513661	7.8691128	0.3195452	0.8482237	No significant change
hsa-miR-138-5p	1.746835	2.9430173	0.3222519	0.8519453	No significant change
hsa-miR-127-5p	1.3399919	1.9387115	0.3238221	0.8526446	No significant change
hsa-miR-6767-5p	–1.215424	2.6648708	0.3260874	0.8551609	No significant change
hsa-miR-378g	–0.675223	4.9405071	0.3296351	0.8602968	No significant change
hsa-miR-1291	–1.052421	3.0656529	0.3314369	0.8602968	No significant change
hsa-miR-340-5p	0.5921506	7.1447921	0.3319982	0.8602968	No significant change
hsa-miR-27b-3p	0.5606084	9.2327937	0.3354214	0.865732	No significant change
hsa-miR-133a-3p	–1.010586	2.795073	0.3382356	0.8695584	No significant change
hsa-miR-3180-3p	–0.846583	3.8598008	0.3411122	0.8701843	No significant change
hsa-miR-3180	–0.846582	3.8598008	0.3411442	0.8701843	No significant change
hsa-miR-548f-3p	1.0661786	3.2749608	0.3481526	0.8846056	No significant change
hsa-miR-454-3p	1.5722845	1.874925	0.3527671	0.8870067	No significant change
hsa-miR-3605-5p	0.8221827	3.5829053	0.3528511	0.8870067	No significant change
hsa-miR-3656	0.9575566	6.401001	0.3541452	0.8870067	No significant change
hsa-miR-200c-3p	–0.712289	4.39608	0.3550301	0.8870067	No significant change
hsa-miR-4668-5p	1.392088	1.6240717	0.3558894	0.8870067	No significant change
hsa-miR-125a-3p	0.5399649	5.1715031	0.3592955	0.8870821	No significant change
hsa-miR-4449	1.5954564	1.9320423	0.3594572	0.8870821	No significant change
hsa-miR-409-3p	–0.543216	9.7619462	0.3613628	0.8870821	No significant change
hsa-miR-191-5p	–0.433838	10.619917	0.3616148	0.8870821	No significant change
hsa-miR-378b	–0.880843	2.2021448	0.363823	0.8870821	No significant change
hsa-miR-7704	–0.984122	3.3957706	0.3641425	0.8870821	No significant change
hsa-miR-221-5p	–1.002764	3.4954176	0.3673952	0.8870821	No significant change
hsa-miR-25-5p	–0.493149	5.6091814	0.3679407	0.8870821	No significant change
hsa-miR-425-3p	0.541883	5.3104613	0.3681459	0.8870821	No significant change
hsa-miR-4533	–1.760573	2.1254106	0.3704092	0.8888605	No significant change
hsa-miR-933	–0.959165	2.3375305	0.3753003	0.8888605	No significant change
hsa-let-7a-5p	0.6497322	12.975814	0.3753405	0.8888605	No significant change
hsa-miR-4470	–0.892524	2.8122058	0.37695	0.8888605	No significant change
hsa-miR-2278	–1.766699	1.8778793	0.3770478	0.8888605	No significant change
hsa-miR-486-5p	–0.457939	16.686046	0.3832073	0.8888605	No significant change
hsa-miR-3940-3p	–1.408048	1.6608732	0.3832843	0.8888605	No significant change
hsa-miR-3187-3p	–0.892137	2.5921695	0.3855747	0.8888605	No significant change
hsa-miR-4497	0.7392705	3.6014612	0.3870867	0.8888605	No significant change
hsa-miR-199b-5p	0.9836411	3.0620057	0.3880651	0.8888605	No significant change
hsa-miR-619-5p	0.7914885	3.6930173	0.3919101	0.8888605	No significant change
hsa-miR-486-3p	–0.527163	9.2947109	0.3922085	0.8888605	No significant change
hsa-miR-877-5p	0.887142	2.7835525	0.3936675	0.8888605	No significant change
hsa-miR-26a-5p	–0.438526	12.034981	0.3941645	0.8888605	No significant change
hsa-miR-130a-3p	0.7931839	3.452487	0.3962262	0.8888605	No significant change
hsa-miR-5189-3p	–1.877746	1.5850335	0.3979336	0.8888605	No significant change
hsa-miR-219a-1-3p	0.6166556	3.9289159	0.3985645	0.8888605	No significant change
hsa-miR-3613-5p	1.0486812	3.4353781	0.3990407	0.8888605	No significant change
hsa-miR-6868-3p	1.5784115	1.83275	0.3993111	0.8888605	No significant change
hsa-miR-4326	0.5059312	4.695875	0.3996936	0.8888605	No significant change
hsa-miR-142-3p	–0.539992	5.2761115	0.3999501	0.8888605	No significant change
hsa-miR-188-5p	–1.772054	1.8330291	0.4003739	0.8888605	No significant change
hsa-miR-628-3p	–0.815403	3.5364941	0.4020194	0.8888605	No significant change
hsa-miR-3163	–1.772486	1.7336399	0.4039195	0.8888605	No significant change
hsa-miR-6734-5p	–0.785277	2.7896561	0.4039329	0.8888605	No significant change
hsa-miR-450a-2-3p	1.4317292	1.7596501	0.404995	0.8888605	No significant change
hsa-miR-181a-2-3p	0.5075986	5.4332687	0.4073637	0.8888605	No significant change
hsa-miR-95-3p	0.8544531	3.1519562	0.4083902	0.8888605	No significant change
hsa-miR-197-3p	–0.449493	5.8276489	0.4095343	0.8888605	No significant change
hsa-let-7d-3p	–0.56787	8.8293206	0.409823	0.8888605	No significant change
hsa-miR-1343-3p	–0.946581	2.5133219	0.411081	0.8888605	No significant change
hsa-miR-28-5p	–0.621388	4.630008	0.4174868	0.8997323	No significant change
hsa-miR-4746-5p	–0.486839	4.7626153	0.4212221	0.9022778	No significant change
hsa-miR-766-5p	–0.737694	3.3727211	0.422033	0.9022778	No significant change
hsa-miR-215-5p	–0.480604	7.1161766	0.4249566	0.9022778	No significant change
hsa-miR-5584-5p	–1.379773	1.7846595	0.425009	0.9022778	No significant change
hsa-let-7e-5p	–0.46396	5.7291892	0.4267814	0.9022778	No significant change
hsa-miR-204-5p	1.0562679	3.1446046	0.4280874	0.9022778	No significant change
hsa-miR-505-3p	1.0115324	2.2007987	0.4311119	0.9022778	No significant change
hsa-miR-3960	–0.820323	3.0252496	0.4312514	0.9022778	No significant change
hsa-miR-769-5p	0.4131347	6.328532	0.434183	0.9022778	No significant change
hsa-miR-574-5p	0.5165305	4.4120608	0.4346184	0.9022778	No significant change
hsa-miR-4745-5p	1.1035172	1.8834507	0.4351774	0.9022778	No significant change
hsa-miR-6826-5p	–1.549823	1.6679906	0.4374285	0.9022778	No significant change
hsa-miR-6873-3p	–1.854291	1.8434449	0.4375316	0.9022778	No significant change
hsa-miR-1255a	–1.492719	1.8463584	0.4389187	0.9022778	No significant change
hsa-miR-196a-5p	1.5897358	1.7211208	0.4393941	0.9022778	No significant change
hsa-miR-541-3p	1.6596473	1.537037	0.4433141	0.9048498	No significant change
hsa-miR-411-5p	–1.076507	2.2087415	0.4435109	0.9048498	No significant change
hsa-miR-5010-3p	–0.883236	2.7159418	0.4448037	0.9048498	No significant change
hsa-miR-627-5p	–0.918525	3.6252789	0.4466178	0.9057188	No significant change
hsa-miR-1285-3p	0.3945564	7.1830884	0.4517263	0.9132424	No significant change
hsa-miR-190b	–0.963908	2.677174	0.4545007	0.9144084	No significant change
hsa-miR-100-5p	0.4200413	9.5921916	0.4560086	0.9144084	No significant change
hsa-miR-361-5p	0.535013	4.8083803	0.4565041	0.9144084	No significant change
hsa-miR-5187-5p	–0.867499	2.9017873	0.4591533	0.9148355	No significant change
hsa-miR-147b	–0.589351	4.4667779	0.4623371	0.9148355	No significant change
hsa-miR-937-3p	–0.974385	1.9353914	0.464301	0.9148355	No significant change
hsa-miR-6510-3p	1.0174261	1.7619231	0.4644156	0.9148355	No significant change
hsa-miR-939-5p	–0.697256	4.0995899	0.4651244	0.9148355	No significant change
hsa-miR-378h	–0.791617	2.3260691	0.4658731	0.9148355	No significant change
hsa-miR-217	1.3021498	1.9050108	0.4665241	0.9148355	No significant change
hsa-miR-7641	–0.468029	4.9366952	0.4690113	0.9154936	No significant change
hsa-miR-548av-3p	0.47114	6.252222	0.4733518	0.9154936	No significant change
hsa-miR-6741-5p	0.5949207	4.1714329	0.475308	0.9154936	No significant change
hsa-miR-654-5p	–0.464514	4.8400168	0.4763995	0.9154936	No significant change
hsa-miR-200b-3p	0.7091144	5.0107698	0.4766057	0.9154936	No significant change
hsa-miR-1255b-5p	–0.451304	5.6056901	0.4779232	0.9154936	No significant change
hsa-miR-491-5p	0.9313127	2.2497678	0.4789445	0.9154936	No significant change
hsa-miR-25-3p	0.3489736	14.27837	0.4791003	0.9154936	No significant change
hsa-miR-941	–0.409436	8.2614403	0.480218	0.9154936	No significant change
hsa-miR-1278	0.7481435	2.8317147	0.4808795	0.9154936	No significant change
hsa-miR-136-3p	1.1333558	2.080222	0.4856282	0.9218466	No significant change
hsa-miR-651-5p	0.8296741	2.8857899	0.4893942	0.9261983	No significant change
hsa-miR-2115-5p	–0.654134	3.8568241	0.4947184	0.9261983	No significant change
hsa-miR-125b-1-3p	–0.710074	2.5821846	0.4951871	0.9261983	No significant change
hsa-miR-432-5p	0.519459	5.7732312	0.4973734	0.9261983	No significant change
hsa-miR-370-3p	–0.449291	8.5231489	0.4995561	0.9261983	No significant change
hsa-miR-30e-3p	0.4303092	6.2012746	0.5015477	0.9261983	No significant change
hsa-miR-4664-3p	0.6964219	2.9212932	0.5026916	0.9261983	No significant change
hsa-miR-199b-3p	0.3983955	7.7412834	0.5028777	0.9261983	No significant change
hsa-miR-199a-3p	0.3983957	7.7412834	0.5029159	0.9261983	No significant change
hsa-miR-3135a	0.84727	2.265136	0.503214	0.9261983	No significant change
hsa-miR-200b-5p	–1.018736	2.4345379	0.5041833	0.9261983	No significant change
hsa-miR-485-5p	–0.452521	5.3178514	0.5057817	0.9261983	No significant change
hsa-miR-7854-3p	0.5668372	4.1261832	0.5063595	0.9261983	No significant change
hsa-miR-6500-3p	1.8457938	1.5789995	0.5102098	0.9267524	No significant change
hsa-miR-342-5p	–0.371101	7.9757891	0.5117793	0.9267524	No significant change
hsa-miR-4429	–0.486907	6.5545003	0.5128256	0.9267524	No significant change
hsa-miR-106b-3p	0.2971658	9.6962724	0.5135598	0.9267524	No significant change
hsa-miR-1468-5p	–0.43149	4.4314904	0.5138861	0.9267524	No significant change
hsa-miR-192-5p	–0.389268	10.907	0.5151779	0.9267524	No significant change
hsa-miR-30b-3p	–0.748655	2.2392432	0.5199714	0.9326946	No significant change
hsa-miR-200a-3p	0.6084755	6.0034314	0.5225511	0.9326946	No significant change
hsa-miR-99b-3p	0.4425954	5.2962311	0.5242053	0.9326946	No significant change
hsa-miR-125b-2-3p	–0.495967	4.2076181	0.5252565	0.9326946	No significant change
hsa-miR-128-3p	–0.413722	11.383902	0.5262359	0.9326946	No significant change
hsa-miR-92b-3p	–0.432807	5.4786538	0.527051	0.9326946	No significant change
hsa-miR-143-5p	0.6269091	3.0870524	0.5297608	0.9335995	No significant change
hsa-miR-3911	0.9320473	2.7415764	0.5304218	0.9335995	No significant change
hsa-miR-103a-3p	0.3054391	10.354731	0.532489	0.933819	No significant change
hsa-miR-30c-2-3p	0.8425485	2.1752655	0.5354484	0.933819	No significant change
hsa-miR-495-3p	0.5291727	5.4675532	0.5364552	0.933819	No significant change
hsa-miR-26a-2-3p	1.5198874	1.6029344	0.537599	0.933819	No significant change
hsa-miR-126-3p	–0.307273	12.413148	0.5394743	0.933819	No significant change
hsa-miR-3682-3p	0.8143584	2.1060439	0.5407325	0.933819	No significant change
hsa-miR-4448	–0.876161	1.5648166	0.5432524	0.933819	No significant change
hsa-miR-132-5p	0.4250566	4.3840081	0.5436233	0.933819	No significant change
hsa-miR-93-3p	–0.591315	2.8480514	0.5445439	0.933819	No significant change
hsa-miR-32-3p	1.4328408	1.761357	0.5497012	0.933819	No significant change
hsa-miR-20a-5p	0.3778838	7.2058472	0.5502776	0.933819	No significant change
hsa-let-7i-3p	–1.322239	1.8790777	0.5505762	0.933819	No significant change
hsa-miR-329-3p	1.0060924	1.8428753	0.5517286	0.933819	No significant change
hsa-miR-493-5p	1.0914151	2.0729394	0.5527974	0.933819	No significant change
hsa-miR-6878-5p	–0.96207	1.8275946	0.5547283	0.933819	No significant change
hsa-miR-151a-3p	–0.27039	15.042613	0.5547518	0.933819	No significant change
hsa-miR-92a-1-5p	–0.931699	2.2804479	0.5563017	0.933819	No significant change
hsa-miR-4660	0.9567759	2.2283856	0.5576035	0.933819	No significant change
hsa-miR-4753-5p	0.8188679	2.3354297	0.5577173	0.933819	No significant change
hsa-miR-340-3p	–0.525408	4.2639904	0.5619548	0.9344595	No significant change
hsa-miR-34c-5p	–0.909724	1.6655184	0.5621801	0.9344595	No significant change
hsa-miR-10b-3p	0.4665191	4.8883603	0.5623929	0.9344595	No significant change
hsa-miR-4661-5p	0.5212449	3.0750091	0.5707537	0.9441812	No significant change
hsa-miR-32-5p	0.5611341	3.1275875	0.5711357	0.9441812	No significant change
hsa-miR-452-5p	–0.358746	6.0219301	0.5733542	0.9444801	No significant change
hsa-miR-548j-3p	–0.753882	2.6190697	0.5742183	0.9444801	No significant change
hsa-miR-148b-5p	–0.665622	2.4664456	0.5756555	0.9444801	No significant change
hsa-miR-1180-3p	–0.27482	9.0281703	0.5774878	0.9448464	No significant change
hsa-miR-17-3p	–0.975126	1.8903205	0.5787727	0.9448464	No significant change
hsa-miR-3127-5p	0.5802173	2.6355728	0.5831993	0.9496986	No significant change
hsa-miR-195-5p	0.7432878	2.5018688	0.5882275	0.9555039	No significant change
hsa-miR-6515-5p	0.480945	3.6303755	0.5977744	0.9686022	No significant change
hsa-miR-1294	0.3463651	4.9323371	0.6005949	0.9707635	No significant change
hsa-miR-6514-5p	0.6198655	2.4006283	0.6028692	0.9720336	No significant change
hsa-miR-7850-5p	–1.024476	2.1457357	0.604671	0.9725373	No significant change
hsa-miR-132-3p	–0.523232	3.0640587	0.6091789	0.9773803	No significant change
hsa-miR-5581-3p	–1.118694	1.7023392	0.6108762	0.9777013	No significant change
hsa-miR-6786-3p	–0.820001	1.8540161	0.6153652	0.9824779	No significant change
hsa-miR-24-1-5p	–0.588733	2.4993969	0.617275	0.9831234	No significant change
hsa-miR-296-3p	0.6198184	2.7034605	0.6200719	0.9851751	No significant change
hsa-miR-598-3p	0.6728758	2.2566853	0.6236678	0.9884833	No significant change
hsa-miR-4646-5p	–0.540847	2.2912729	0.6262776	0.9902161	No significant change
hsa-miR-4440	–0.590112	2.2793566	0.6389004	0.998528	No significant change
hsa-miR-1273 h-5p	–0.423921	3.790786	0.6391411	0.998528	No significant change
hsa-miR-576-3p	–0.237687	8.7340672	0.6404048	0.998528	No significant change
hsa-miR-4516	0.5384799	1.8270786	0.6444029	0.998528	No significant change
hsa-miR-28-3p	–0.216384	9.2362065	0.6463092	0.998528	No significant change
hsa-miR-140-5p	0.3691516	4.3450804	0.6475597	0.998528	No significant change
hsa-miR-4781-3p	–0.766654	1.9600532	0.6478883	0.998528	No significant change
hsa-miR-320b	–0.318763	13.64376	0.6490834	0.998528	No significant change
hsa-miR-4726-5p	–1.115035	1.9321852	0.6506376	0.998528	No significant change
hsa-miR-615-3p	0.4995548	5.2957599	0.6513457	0.998528	No significant change
hsa-miR-4437	1.1238567	1.7013377	0.6521391	0.998528	No significant change
hsa-miR-185-3p	–0.275583	5.4041765	0.6547088	0.998528	No significant change
hsa-miR-1250-5p	–0.460275	3.1292287	0.6572677	0.998528	No significant change
hsa-miR-532-5p	0.2024342	10.463505	0.6572883	0.998528	No significant change
hsa-miR-3620-5p	0.5327481	2.1730518	0.6581288	0.998528	No significant change
hsa-miR-6862-5p	0.7339734	1.6578542	0.6583556	0.998528	No significant change
hsa-miR-339-5p	0.3599348	3.5420369	0.6601304	0.998528	No significant change
hsa-miR-193b-3p	0.8355395	1.8348219	0.6603539	0.998528	No significant change
hsa-miR-24-3p	0.2119127	12.483615	0.6619422	0.998528	No significant change
hsa-miR-361-3p	0.1967387	8.9290252	0.6630333	0.998528	No significant change
hsa-miR-4487	–1.150809	1.5697953	0.6636738	0.998528	No significant change
hsa-miR-548 am-3p	0.5151226	2.9610665	0.6672588	0.998528	No significant change
hsa-miR-335-3p	0.4041049	3.4163132	0.6699615	0.998528	No significant change
hsa-miR-484	0.229637	8.2303032	0.6701036	0.998528	No significant change
hsa-miR-320d	0.3203703	8.7720833	0.6708687	0.998528	No significant change
hsa-let-7d-5p	0.2711084	9.2664515	0.6716954	0.998528	No significant change
hsa-miR-196b-5p	0.4329791	2.7583364	0.6767531	0.998528	No significant change
hsa-miR-27b-5p	0.558564	2.7912571	0.6788834	0.998528	No significant change
hsa-miR-1287-5p	0.3675621	3.7248861	0.6808377	0.998528	No significant change
hsa-miR-92a-3p	–0.252647	13.837765	0.6825184	0.998528	No significant change
hsa-miR-1273c	–0.552522	2.4781665	0.6829609	0.998528	No significant change
hsa-miR-22-3p	–0.181389	12.447027	0.6853361	0.998528	No significant change
hsa-miR-766-3p	0.5614117	2.0406306	0.6855555	0.998528	No significant change
hsa-miR-6503-3p	–0.300207	3.7972393	0.6860794	0.998528	No significant change
hsa-miR-1285-5p	0.9950226	1.6454397	0.6864892	0.998528	No significant change
hsa-miR-181c-3p	0.4292827	2.6901574	0.6865836	0.998528	No significant change
hsa-miR-4467	0.593421	2.5365632	0.6902773	1	No significant change
hsa-miR-642a-3p	–0.475432	3.218189	0.6940372	1	No significant change
hsa-miR-3605-3p	–0.659132	2.4770723	0.6959227	1	No significant change
hsa-miR-3120-3p	–0.325219	3.1488579	0.6986896	1	No significant change
hsa-miR-1273d	–0.390783	2.8524222	0.7006583	1	No significant change
hsa-miR-222-3p	0.2087115	8.733134	0.7030174	1	No significant change
hsa-miR-181b-5p	0.2230656	7.8256168	0.7093748	1	No significant change
hsa-miR-4508	0.2850097	10.424708	0.7104169	1	No significant change
hsa-miR-548u	–0.564302	1.9814485	0.7105777	1	No significant change
hsa-miR-99b-5p	0.1950643	9.4466971	0.7123201	1	No significant change
hsa-miR-1261	0.6235688	1.6884782	0.7126422	1	No significant change
hsa-miR-381-3p	–0.230675	5.2889809	0.7160037	1	No significant change
hsa-miR-504-5p	0.6595251	1.840255	0.7163579	1	No significant change
hsa-let-7f-1-3p	–0.456075	2.6797566	0.7247934	1	No significant change
hsa-miR-18a-3p	–0.443758	3.3336327	0.7260352	1	No significant change
hsa-miR-618	0.4127825	2.9891759	0.726983	1	No significant change
hsa-miR-3138	–0.74913	1.8268181	0.7309819	1	No significant change
hsa-miR-328-3p	–0.172131	6.7513695	0.7320352	1	No significant change
hsa-miR-6806-3p	0.3706707	3.2665815	0.7329487	1	No significant change
hsa-miR-144-3p	–0.190136	5.9284579	0.7339561	1	No significant change
hsa-miR-1270	0.520736	2.0035059	0.7360031	1	No significant change
hsa-miR-4659b-3p	–0.917316	1.5945608	0.736333	1	No significant change
hsa-miR-514a-3p	0.6644575	1.7627173	0.7364687	1	No significant change
hsa-miR-4654	–0.639538	2.2814597	0.7428239	1	No significant change
hsa-miR-7976	0.2280299	4.0552938	0.7431302	1	No significant change
hsa-miR-873-3p	0.2994708	4.2232817	0.7461015	1	No significant change
hsa-miR-424-3p	0.1711323	7.213438	0.7466318	1	No significant change
hsa-miR-942-3p	–0.57066	2.2759455	0.7520299	1	No significant change
hsa-miR-214-5p	0.431421	2.5325743	0.7535177	1	No significant change
hsa-miR-610	–0.383532	2.7463828	0.75393	1	No significant change
hsa-miR-186-5p	–0.146939	10.210479	0.7553595	1	No significant change
hsa-miR-629-5p	0.1695189	11.264791	0.7570134	1	No significant change
hsa-miR-205-5p	–0.455482	2.2163267	0.7575052	1	No significant change
hsa-miR-1538	–0.377836	2.4773178	0.7581975	1	No significant change
hsa-miR-4742-3p	–0.574425	1.9656415	0.7616509	1	No significant change
hsa-miR-152-3p	–0.174723	7.8755973	0.7639043	1	No significant change
hsa-miR-1260b	–0.231988	4.0258788	0.764817	1	No significant change
hsa-miR-191-3p	–0.25857	3.61125	0.7662529	1	No significant change
hsa-miR-1290	0.1721372	8.2902743	0.7672001	1	No significant change
hsa-miR-3158-3p	–0.150834	10.637015	0.7672692	1	No significant change
hsa-miR-128-1-5p	0.5000403	2.0766789	0.7682668	1	No significant change
hsa-miR-4286	0.5162647	1.8443175	0.7748452	1	No significant change
hsa-miR-335-5p	0.1935597	5.8316106	0.7760272	1	No significant change
hsa-miR-210-3p	–0.196257	5.3267414	0.7767807	1	No significant change
hsa-miR-26b-5p	0.1781976	7.6648112	0.779013	1	No significant change
hsa-miR-7-5p	0.1337923	11.093445	0.7829657	1	No significant change
hsa-miR-3691-5p	0.379601	2.1907458	0.7857847	1	No significant change
hsa-miR-99a-5p	–0.150372	13.569867	0.7888686	1	No significant change
hsa-miR-183-5p	0.1712702	8.4813048	0.7917523	1	No significant change
hsa-miR-6821-5p	–0.492681	1.8236572	0.7997148	1	No significant change
hsa-miR-6750-5p	–0.256561	2.9791013	0.8027427	1	No significant change
hsa-miR-18a-5p	0.5312005	1.9085935	0.8039216	1	No significant change
hsa-miR-3679-5p	–0.50048	1.9657891	0.8070612	1	No significant change
hsa-miR-345-5p	–0.126043	7.1103577	0.8075758	1	No significant change
hsa-miR-155-5p	0.1897688	5.3261139	0.8080494	1	No significant change
hsa-miR-659-5p	–0.176787	4.4825036	0.8089872	1	No significant change
hsa-miR-4772-5p	–0.384025	2.2172399	0.8123832	1	No significant change
hsa-miR-576-5p	–0.317823	3.4446128	0.8160607	1	No significant change
hsa-miR-6829-5p	0.6573233	1.6803528	0.8162284	1	No significant change
hsa-miR-5189-5p	–0.264712	3.6969312	0.8197824	1	No significant change
hsa-miR-4797-3p	0.4977217	1.5706456	0.820753	1	No significant change
hsa-miR-378d	0.1598743	6.4744816	0.8221808	1	No significant change
hsa-miR-151a-5p	–0.18985	3.7719524	0.8231726	1	No significant change
hsa-miR-320c	–0.159486	10.917939	0.8235317	1	No significant change
hsa-miR-106b-5p	–0.138543	5.3341027	0.824224	1	No significant change
hsa-miR-379-5p	0.222008	4.5838907	0.8261351	1	No significant change
hsa-miR-20b-5p	–0.13832	4.8649519	0.8270214	1	No significant change
hsa-miR-1273f	0.2724247	2.587597	0.8292921	1	No significant change
hsa-let-7b-5p	–0.10784	13.325142	0.8299265	1	No significant change
hsa-miR-3140-3p	–0.352147	2.0932723	0.8308651	1	No significant change
hsa-miR-145-5p	0.1794492	4.3511282	0.8321114	1	No significant change
hsa-miR-548ap-5p	0.2600483	2.013502	0.8325445	1	No significant change
hsa-miR-181a-3p	–0.210737	3.0554496	0.8333932	1	No significant change
hsa-miR-378i	–0.12108	8.7999496	0.8356999	1	No significant change
hsa-miR-3199	–0.464373	1.9923827	0.8375008	1	No significant change
hsa-miR-3127-3p	–0.622897	1.8005486	0.839874	1	No significant change
hsa-miR-429	–0.349153	2.5510879	0.8453863	1	No significant change
hsa-miR-374a-5p	0.2264504	3.08939	0.8493638	1	No significant change
hsa-miR-382-5p	0.1095212	6.9416982	0.8513419	1	No significant change
hsa-miR-6761-5p	0.623679	1.5495384	0.851564	1	No significant change
hsa-miR-6875-5p	0.5211668	1.9400319	0.8515798	1	No significant change
hsa-miR-423-3p	–0.081611	11.872407	0.8551171	1	No significant change
hsa-miR-6087	0.5822617	1.6075862	0.8554071	1	No significant change
hsa-miR-6882-5p	0.5191495	1.5787126	0.8563317	1	No significant change
hsa-miR-3196	–0.549764	1.5610905	0.8597365	1	No significant change
hsa-miR-96-5p	0.2017521	3.5027927	0.8604698	1	No significant change
hsa-miR-339-3p	–0.101156	7.3890387	0.8610593	1	No significant change
hsa-miR-30e-5p	–0.080126	9.4771337	0.8619278	1	No significant change
hsa-miR-23a-5p	–0.152271	3.5446331	0.8653128	1	No significant change
hsa-miR-4657	0.2977558	2.2053827	0.8655121	1	No significant change
hsa-miR-4741	–0.333654	1.7772274	0.8678381	1	No significant change
hsa-miR-574-3p	–0.137952	4.5991981	0.8685275	1	No significant change
hsa-miR-493-3p	–0.1203	5.6154489	0.869264	1	No significant change
hsa-miR-455-5p	–0.157462	4.0515121	0.8712733	1	No significant change
hsa-miR-4747-5p	–0.661522	1.7532446	0.8736084	1	No significant change
hsa-miR-3179	0.6739758	1.5372134	0.8739323	1	No significant change
hsa-miR-374a-3p	0.3015378	1.7177352	0.8780622	1	No significant change
hsa-miR-378a-5p	0.2966664	2.4421476	0.8791134	1	No significant change
hsa-miR-760	–0.093872	5.9505312	0.8803434	1	No significant change
hsa-miR-4639-5p	–0.473142	1.6020946	0.8813641	1	No significant change
hsa-miR-3176	0.1416603	2.633019	0.8829953	1	No significant change
hsa-miR-3124-5p	–0.173389	3.1259251	0.8844519	1	No significant change
hsa-miR-625-3p	–0.107002	6.3989775	0.8845203	1	No significant change
hsa-miR-3150b-3p	–0.09592	5.0180538	0.8850272	1	No significant change
hsa-miR-4466	–0.251913	1.8275556	0.8857244	1	No significant change
hsa-miR-106a-5p	0.1404881	3.9283878	0.8875824	1	No significant change
hsa-miR-24-2-5p	–0.076083	5.9103213	0.8884291	1	No significant change
hsa-miR-1254	0.1420986	3.1668722	0.889156	1	No significant change
hsa-miR-10a-5p	–0.092127	11.262034	0.8892013	1	No significant change
hsa-miR-17-5p	–0.078709	6.5255191	0.8896858	1	No significant change
hsa-miR-330-5p	–0.082862	5.9135167	0.889776	1	No significant change
hsa-miR-3074-5p	0.2956317	1.9012105	0.8900359	1	No significant change
hsa-miR-136-5p	0.1804324	3.1208797	0.8961827	1	No significant change
hsa-miR-324-3p	–0.133559	3.0058811	0.8999955	1	No significant change
hsa-miR-550a-3-5p	0.0642257	6.5796374	0.9014026	1	No significant change
hsa-miR-26b-3p	–0.307955	2.0010125	0.9021122	1	No significant change
hsa-miR-4498	–0.334558	1.7491314	0.9061182	1	No significant change
hsa-miR-331-3p	0.2704766	2.0127679	0.9077998	1	No significant change
hsa-miR-579-5p	–0.245458	2.6388852	0.9080854	1	No significant change
hsa-miR-195-3p	0.1067741	4.2095303	0.9104302	1	No significant change
hsa-miR-365b-5p	0.2707665	1.9595275	0.9105269	1	No significant change
hsa-miR-3909	–0.307887	1.9703924	0.9147102	1	No significant change
hsa-miR-146a-5p	–0.049813	12.137534	0.915202	1	No significant change
hsa-miR-3064-5p	0.2325321	2.1106126	0.9163336	1	No significant change
hsa-miR-625-5p	0.3627439	1.8957884	0.9165356	1	No significant change
hsa-miR-499a-5p	0.0695846	4.7989112	0.9183591	1	No significant change
hsa-miR-548q	–0.43038	1.8164982	0.9198613	1	No significant change
hsa-miR-5009-5p	–0.085235	3.9249393	0.9211642	1	No significant change
hsa-miR-4473	0.1809776	1.9647736	0.9229154	1	No significant change
hsa-miR-616-3p	–0.148683	2.3564493	0.9251134	1	No significant change
hsa-miR-1260a	0.1245625	3.3867094	0.9268289	1	No significant change
hsa-miR-127-3p	–0.057308	7.7098832	0.9280109	1	No significant change
hsa-miR-10a-3p	0.201966	2.4165085	0.9285191	1	No significant change
hsa-miR-942-5p	–0.050538	5.7554159	0.9304011	1	No significant change
hsa-miR-3150a-5p	–0.248033	2.1840462	0.9333478	1	No significant change
hsa-miR-5010-5p	–0.189689	2.350888	0.9345151	1	No significant change
hsa-miR-19a-3p	0.0668718	5.5469139	0.9349623	1	No significant change
hsa-miR-4647	0.1021053	3.7580773	0.9361456	1	No significant change
hsa-miR-30c-5p	–0.0508	6.8428024	0.937609	1	No significant change
hsa-miR-221-3p	0.0399429	8.2407437	0.9392216	1	No significant change
hsa-miR-30d-5p	–0.03734	12.788792	0.9417539	1	No significant change
hsa-miR-200a-5p	0.2100791	2.914263	0.9417815	1	No significant change
hsa-miR-4732-5p	–0.049304	6.2671787	0.9429188	1	No significant change
hsa-miR-372-3p	0.1224369	3.8880673	0.9438989	1	No significant change
hsa-miR-93-5p	–0.03724	7.579707	0.9448046	1	No significant change
hsa-miR-550a-5p	0.0395062	6.1547169	0.9474948	1	No significant change
hsa-miR-21-5p	–0.030495	11.73524	0.947789	1	No significant change
hsa-miR-342-3p	0.0479521	4.6590461	0.9550105	1	No significant change
hsa-miR-23b-5p	–0.068261	3.8807056	0.95638	1	No significant change
hsa-miR-3190-3p	0.1326734	2.9480806	0.9571401	1	No significant change
hsa-miR-363-5p	0.1331389	2.9921604	0.9584856	1	No significant change
hsa-miR-874-3p	–0.074864	3.6263968	0.9593245	1	No significant change
hsa-miR-4755-3p	–0.080479	3.1370278	0.9604028	1	No significant change
hsa-miR-1-3p	–0.036418	7.1798175	0.9632119	1	No significant change
hsa-miR-126-5p	–0.065305	4.0084407	0.9647803	1	No significant change
hsa-miR-652-3p	–0.029345	5.9122246	0.9663523	1	No significant change
hsa-miR-129-5p	–0.028294	6.9821323	0.9675328	1	No significant change
hsa-miR-16-2-3p	–0.027523	8.811907	0.9683208	1	No significant change
hsa-miR-1306-3p	–0.031442	5.7717178	0.970366	1	No significant change
hsa-miR-92b-5p	–0.04648	4.4099962	0.9724563	1	No significant change
hsa-miR-194-5p	0.022113	7.9164417	0.9727306	1	No significant change
hsa-let-7b-3p	0.0437895	3.4838939	0.9734918	1	No significant change
hsa-miR-130b-3p	–0.025812	4.1504386	0.9771869	1	No significant change
hsa-miR-584-5p	–0.015047	9.1985501	0.9774408	1	No significant change
hsa-miR-151b	0.0243652	4.2112358	0.9787314	1	No significant change
hsa-miR-181a-5p	–0.010282	10.939446	0.9820032	1	No significant change
hsa-miR-6852-5p	0.018814	4.5947672	0.9862641	1	No significant change
hsa-miR-425-5p	0.0062513	8.9639875	0.9896049	1	No significant change
hsa-miR-181d-5p	–0.008651	5.0182536	1	1	No significant change
hsa-miR-516b-5p	–0.102628	2.8190711	1	1	No significant change
hsa-miR-641	–0.097843	2.2703868	1	1	No significant change
hsa-miR-1273a	–0.342187	1.5409936	1	1	No significant change
hsa-miR-548j-5p	0.2238124	1.9356194	1	1	No significant change
hsa-miR-548az-5p	0.132175	2.2329598	1	1	No significant change
hsa-miR-371b-5p	0.3474716	1.6005097	1	1	No significant change
hsa-miR-6716-3p	0.3315585	1.5923398	1	1	No significant change
hsa-miR-4306	–0.328855	1.5904479	1	1	No significant change
hsa-miR-6839-5p	0.2973405	1.6928111	1	1	No significant change
hsa-miR-6774-5p	–0.253602	1.5400938	1	1	No significant change
hsa-miR-6818-5p	–0.234782	1.882618	1	1	No significant change
hsa-miR-6781-5p	0.2234265	1.5361868	1	1	No significant change
hsa-miR-4800-5p	0.2154358	2.0145414	1	1	No significant change
hsa-miR-365a-5p	0.1827695	1.7779599	1	1	No significant change
hsa-miR-500b-3p	0.1643585	2.0396018	1	1	No significant change
hsa-miR-581	0.1637864	1.9969262	1	1	No significant change
hsa-miR-6501-5p	0.1422351	1.7067184	1	1	No significant change
hsa-miR-494-3p	–0.131872	1.772495	1	1	No significant change
hsa-miR-556-5p	–0.13052	1.7384358	1	1	No significant change
hsa-miR-6780a-5p	0.1235197	1.982604	1	1	No significant change
hsa-miR-6815-5p	–0.074996	2.4337051	1	1	No significant change
hsa-miR-5096	0.0728714	2.3294423	1	1	No significant change
hsa-miR-5003-3p	–0.070553	2.0370795	1	1	No significant change
hsa-miR-4724-5p	0.0690548	1.7048819	1	1	No significant change
hsa-miR-431-5p	0.0633666	2.2752355	1	1	No significant change
hsa-miR-664b-5p	–0.062252	2.0530893	1	1	No significant change
hsa-miR-3122	–0.060705	1.7377483	1	1	No significant change
hsa-miR-3690	0.0440439	3.0985568	1	1	No significant change
hsa-miR-30c-1-3p	–0.037941	1.8684588	1	1	No significant change
hsa-miR-3675-5p	0.0377325	1.9123711	1	1	No significant change
hsa-miR-6733-5p	–0.024292	1.5948618	1	1	No significant change
hsa-miR-4433b-3p	–0.014877	4.8281722	1	1	No significant change
hsa-miR-412-5p	–0.005298	2.4102135	1	1	No significant change
hsa-miR-29c-5p	–0.001197	2.7045493	1	1	No significant change
hsa-miR-144-5p	–0.000254	4.0263451	1	1	No significant change
hsa-miR-375	–2.907928	10.621211	4.83E-08	3.16E-05	Down
hsa-miR-885-3p	–5.273985	2.4001832	0.0012326	0.2345272	Down
hsa-miR-122-5p	–2.159821	17.22628	0.0025547	0.2345272	Down
hsa-miR-4433b-5p	–2.274328	4.5227663	0.0026641	0.2345272	Down
hsa-miR-4446-3p	–1.711338	4.6900748	0.0070462	0.2367778	Down
hsa-miR-885-5p	–4.134922	2.5015312	0.0084043	0.2494538	Down
hsa-miR-362-5p	–4.25044	2.1184963	0.0100574	0.2623126	Down
hsa-miR-150-3p	–1.462253	6.4066843	0.010493	0.2623126	Down
hsa-miR-3173-5p	–2.254604	2.6784423	0.0169241	0.3683817	Down
hsa-miR-139-3p	–1.429749	6.6299538	0.0182671	0.3847879	Down
hsa-miR-378e	–2.947571	3.6008184	0.0217626	0.4111132	Down
hsa-miR-193b-5p	–1.525996	7.0763764	0.0220352	0.4111132	Down
hsa-miR-4435	–2.038539	2.5392622	0.0239251	0.4123441	Down
hsa-miR-3191-3p	–3.112364	2.2600313	0.0324487	0.4546377	Down
hsa-miR-4665-5p	–1.786367	4.0103789	0.0351945	0.4787914	Down
hsa-miR-1249-3p	–4.366691	1.6361465	0.0367761	0.4900979	Down
hsa-miR-1299	–2.231941	8.5118765	0.0377554	0.4914672	Down
hsa-miR-6884-5p	–1.81515	4.2670509	0.0391367	0.4914672	Down
hsa-miR-1247-5p	–4.90665	1.8784532	0.0407562	0.5021471	Down
hsa-miR-4732-3p	–1.108072	6.6087268	0.04229	0.5113961	Down
hsa-miR-320e	–1.994268	5.4474059	0.0455123	0.5261696	Down
hsa-miR-146b-3p	–1.406413	4.6172767	0.046692	0.5261696	Down
hsa-miR-6511a-5p	–3.261756	2.1336766	0.0467348	0.5261696	Down
hsa-miR-193a-5p	–1.202926	11.155122	0.0489339	0.5415905	Down

**Table 2:** Target genes of the three miRNAs.

Potential targets of hsa-miR-15b-5p	Potential targets of hsa-miR-16-5p	Potential targets of hsa-miR-184	Potential targets of hsa-miR-15b-5p AND hsa-miR-16-5p NOT hsa-miR-184	Potential targets of hsa-miR-15b-5p AND hsa-miR-184 NOT hsa-miR-16-5p	Potential targets of hsa-miR-16-5p AND hsa-miR-184 NOT hsa-miR-15b-5p	Potential targets of hsa-miR-15b-5p AND hsa-miR-16-5p AND hsa-miR-184
CPD	EXT1	STRADA	STAMBPL1	TASOR	CCDC80	RAB30
USP14	RNF157	BCAT2	DCAF12L2	ELP2	LIPH	BCL11A
SETD5	PDLIM7	STX6	CTSC	PI4K2A	OPN3	ZMAT3
SESN1	NCOA6	TLCD3A	RCC2	FAM204A	PHKG2	NEK10
TTPAL	NCS1	FTCD	MCRIP1	CYB561D1	SLC4A8	PPP2R2B
GLS2	UBE2J1	CHST14	GFI1B	NOP9	XRRA1	CDRT1
GALK2	PHACTR4	MAP4K4	CUL3	CTDP1	IST1	BEST3
DCLRE1C	PPM1D	ERVV-1	RAB44	TSHZ2	ZFAND5	MTA3
CRADD	TRIP12	SYNE3	UBE2V1	RAI14	DIXDC1	MPI
TSPYL5	FBXW7	ZNF707	FRMD6	RBBP4	SURF4	TMEM106A
B3GAT1	RPS14	DLX1	CLOCK	CBX5	RAD9B	TMCC3
SNRK	DHX33	RNF38	RAB3IP	TBCD	ZRANB3	SNX19
PRAMEF8	TMEM143	M1AP	STT3A	STK24	RUBCNL	PARP15
AKR1B15	DEPDC1B	HSPA12A	PDK1	JAKMIP3	CCL4L2	TFRC
MBP	IRAK3	MTMR10	HPCAL4	SSBP2	RXRA	DLK1
VASH1	MRTFA	SLC16A3	TTC1	NFATC2	ACP2	TBC1D7
OTUB1	DDX6	EFCAB14	TLE4	SHTN1	CYSLTR2	GPR63
SMURF1	IKZF3	JOSD1	GOT2	CDH19	EZH1	SON
ODF2L	VPS50	RAB21	USP49	DNAJC10	C1orf21	SLC9A7
PTPRJ	SLC4A7	RETSAT	ADD1	CSNK1A1	HNRNPDL	BTBD9
SORD	KIF18B	SHQ1	CALML4	ORAI2	CCDC115	XKR4
VPS9D1	GPR161	QRSL1	RCL1	SCIMP	NFKBID	NOPCHAP1
CORIN	CD274	CYCS	NADK2	IGSF9B	GNB1L	ZNF597
HSPA13	SOCS2	MDM1	CPEB1	AMOTL2	SLC30A7	PANK2
KRR1	MCU	UBR4	POLH	CEP43	CREB1	MOB1B
IL3	PGR	SYT13	TRERF1	ARFGAP1	TMEM183A	SYT9
COX19	PTGS1	PTPA	PRDM5	NCAPH	SNX1	UBE2D3
RPGR	CCDC177	CDC25B	MYO6	FSD2	GJD3	NRP2
CHIC1	KCNMA1	KLRB1	ACER3	GNA13	FBXW8	NCKAP1
AGAP3	SLC39A1	KPNA1	TPD52L1	ADNP	CNTN5	MBD1
PHTF2	VWA2	PLAGL2	ARHGEF12	USP47	MTMR11	AREL1
CD209	DUOXA1	CCL4	SLMAP	FAM107B	BIRC3	KIAA1549L
NR4A1	KCNJ15	SDC1	NKAIN3	XRN1	AK4	RALGPS1
YOD1	ZNF468	ASTN1	MBNL2	EIF4E3	TLR6	SUSD6
DNAI4	GDNF	RAB7A	ARPP19	PTCD2	TMEM154	WAPL
SLC12A2	ZKSCAN8	PCYT1A	DCUN1D1	LYSMD4	RNF150	ZBTB20
TBC1D16	NUP155	TROAP	TGFA	TPD52	FHIP1A	CNNM2
ZBTB18	FBXL7	HMX2	HEMK1	EYA1	SEMA4D	AP5S1
MARCHF8	FRS2	CERT1	PCDH9	KIF6	KSR1	JPH1
AMZ1	EDIL3	CCT3	ACSL4	CECR2	RBFOX2	MS4A7
ZNF75A	LYPLA1	LAPTM5	B3GNT2	CTBP2	SEC14L5	MASP1
SLC2A10	ATP7A	RPGRIP1L	RACGAP1	CERS3	CDC42BPA	CTSH
STPG4	GNA12	TPRA1	PIAS1	DHFR	ZBTB5	KIF3B
THRA	GAPVD1	NHEJ1	STS	TMCC2	ASXL1	GFRA1
CD164L2	CDV3	EN1	ZNF436	TTC39A	IRF2BP1	CD6
BCL6	POLR1B	CHRNA7	OR14J1	SLC9B2	RAB23	ZBTB43
GINS1	RIMS2	LIN52	TRIM56	CD55	KLHL3	TBL1XR1
GRM5	ZNF341	ZC2HC1C	SH3BGRL2	CCL28	GALNT7	SOD2
TCEAL6	GUCD1	PPP1R18	PSD2	ZNF639	GSPT2	FLRT2
EPSTI1	RBM7	PARP9	GPR174	MLEC	UBE2W	ZNF544
YTHDF1	TMEM132B	P2RX6	FOXP1	KRBA2	TXLNG	B4GALT1
EYA3	SUMO3	KLF6	RPL13	LNPK	UBFD1	BRWD1
ZNF217	THSD4	CNDP1	GBP4	NAT9	EIF5A2	RNLS
SMC1A	CENPW	MBTD1	PALM2AKAP2	ZFP90	ABO	SCN3B
GABPB2	AIG1	ZNF517	MACROD2	ARHGEF6	GALNT1	SLC6A4
KLHDC7A	VEGFA	SMIM10L1	IRAG1	SETD7	FMR1	RBM23
DCX	ITPRID2	CEP350	ALKBH3	MRTFB	RAB3B	GLRX
ADAMTS17	STK32A	DIDO1	GIT2	TNFAIP8L3	SLAMF1	TGFBR3
PEX26	DMPK	LPCAT3	YWHAZ	PLCB3	SRPK1	CCNT2
TNNI1	TTC7A	SPP1	CHMP4C	UVSSA	STC1	
FHDC1	PHLPP2	COMMD7	FAM241A	BNC2	PDXK	
GUCY1A1	COPG2	AMPD2	RDH12	ENTPD6	ARL3	
ZNF74	POLDIP2	KLHL36	ABHD2	RPS6KA2	KIF2A	
PRMT8	NBR1	MPZ	GPR26	PLEKHO2	XPR1	
GDAP1L1	HNRNPR	CHFR	SEMA6D	APOL6	ONECUT2	
PEX5L	ZNF417	SMC6	ZNF398	ZNF442	PTBP3	
RNF165	EIF4B	STOML1	ANAPC16	GABARAPL1	PDK3	
RIT1	WNT8A	ABR	MALT1	THAP2	KLC1	
ZNF382	ANKRD42	PAX1	CSNK2A1	PCDHA4	RGS4	
SLC20A2	RAB11FIP4	LIF	PAK5	MRPS5	MANBA	
FGF1	RPL10	GPD1	IL21R	RBM48	TRA2A	
GRIA1	USP6	ZNF451	ORC4	TMEM164	USP25	
HMBS	MED20	ATP5F1A	BTLA	ZBED3	METTL8	
ZNF501	ZNF827	BOD1L2	UBE2I	ZBTB37	CAMK4	
MSH2	PLEKHG3	ITPRID1	MRRF	RSPO3	TACC1	
CLPB	CCNJL	EFTUD2	PEDS1-UBE2V1	ADO	LOC112694756
PKD2L2	TSHZ1	EPS15L1	SERTAD3	B3GALNT1	FAM149A	
SLC9A8	AAMDC	RPS3	PLPP6	STX1B	HECTD4	
NOL10	AGO1	CALCOCO2	GAB1	EXOSC6	IRAK2	
TSN	CHCHD7	PTGES3L	XKR5	CNTNAP5	FXN	
PDPK1	CARD19	ZNF484	SART3	OTUD7A	SLC11A2	
CDX2	SALL4	CDS1	IFT140	MBNL3	HMGXB4	
FAM13A	AK2	SMC2	POU2F1	MTO1	ZXDC	
ZNF211	GMEB1	UNC45B	KIF5C	KCNK10	WTIP	
PSME3	ZNF326	PLEKHB2	NCBP2	RHBDL3	NCBP3	
CREM	ZDHHC23	CTCFL	SIAH1	BACE2	ATXN1	
TBC1D2	KIF1A	ANKRD46	LRRC63	TOR1AIP2	CACNB4	
MGST1	MATN4	G6PC1	PRKAG3	SKA3	TTC39B	
ZNF850	DICER1	RXRB	MGST2	ZNF641	RAB3GAP1
PSMD5	SPHKAP	CDK2AP1	SEMA3A	CCNYL1	PEG10	
CDC42EP3	FCRL2	STOM	SREBF1	ZNF555	MAMLD1	
PSMD11	COG3	KLF7	SMAD2	ST6GALNAC3	GFOD1	
CHD2	SESN2	NFIX	CHEK1	PRICKLE1		
PCED1A	SYT15	ZNF512	GLYCTK	MRPL42		
CALCRL	TSSK6	SEMA4F	UTP25	APBB2		
ITGBL1	C19orf44	PUS7L	NEK6	LSM11		
CEP57L1	SLITRK6	RCN2	SULT1B1	CLVS1		
SZRD1	RTL6	AAGAB	CCDC144A	PGM2L1		
SRGAP2B	TCHP	STK25	KIAA0408	OR7D2		
KIF27	NTPCR	RAB4A	PHACTR2	PSMF1		
SLIT3	PRXL2A	KCNT1	AAK1	GPAT4		
ITPRIP	LCOR	LRRC14	KIF1B	HNF4A		
PRKAR1A	DCTN5	RPS6KB1	SNX13	LDLRAD4		
LZTFL1	PARD6B	CHD4	DENND5A	CUX1		
HOMER1	USP32	BBX	ANAPC13	ZNF778		
ZNF550	CBR4	ELF2	INTU	ZNF605		
WDR72	ZNF587	ZNF891	DCAF8	UBE2L6		
FMN1	SEC22C	POTEI	CNR1	GPRIN3		
ANKRD62	TRIM4	GANAB	GOLGA7	CNNM3		
RFPL4AL1	LMLN	CLCC1	RAB9B	DSTYK		
SDHC	FGF5	LMCD1	ERAP1	GAS7		
SLC38A1	KRAS	PTPN5	RGS3	QKI		
ACVR2A	TPGS1	CARHSP1	CDC37L1	NFASC		
MYBL2	LUZP1	LILRA1	EPB41L4B	RNF213		
RANGAP1	PLEKHG4B	ACTN2	SLC35E3	RPRD1B		
FBXL4	FCRL3	PHYKPL	NUP107	LRRC3B		
REXO4	DNAJB6	RHOH	SPC25	RUBCN		
JRK	KCNG3	COBLL1	TAOK1	NPY4R2		
DPF3	RUNDC3B	MDM2	HOXD11	ADCYAP1R1	
ZNHIT3	AKAP7	MON2	SRPRB	SEC22A		
MAP2K4	MSI2	GPATCH11	PELI2	FBXO3		
SMAP1	CD151	CRNKL1	PROK2	CLUH		
UPK1A	MRPL30	SSTR3	XPNPEP3	ZNF701		
SLC35C2	APOBEC3F	ELN	TMEM38A	CBFA2T2		
MCM8	TNFRSF10B	PHB	TTC13	EIF2AK2		
RFX3	SVIP	HEPH	GALNT12	REEP1		
SSH2	HGSNAT	PYCR1	RBFA	MMS22L		
PPP2R5E	AMER1	OSCAR	CCDC134	EML1		
WFDC1	TLCD4	ARPIN	DLST	ALDH1L2		
ZFP37	PGBD4	MDH2	HSPA4	MTERF4		
FAM243A	METTL15	KCTD14	KPNA4	TRAK1		
H6PD	NATD1	GOLGA8M	NDUFB5	PTCHD4		
ARSL	IP6K1	GOLGA8H	RAD51D	TTBK1		
FAM107A	BEST4	GOLGA8K	SRP19	NCOA2		
HEY1	HYAL1	ZNF343	VSNL1	ZNF276		
TRIB1	ZNF791	BTBD3	ACOX3	NHSL2		
CIBAR1	LNX2	HHLA2	MPDZ	MKLN1		
ZFYVE16	ZDHHC14	ZCCHC17	GPRC5A	CELF2		
VOPP1	KMT2C	SLC66A3	PRDX6	MAPRE2		
FKBP9	RASSF2	KIAA1217	PPARA	BMP10		
LRRC49	TEDDM1	CCNY	CCDC6	FAM131B		
SLC36A4	RDH10	RASGEF1A	KARS1	PTDSS1		
SLC2A14	DCUN1D3	MTSS1	NFIB	MRPL19		
RALGPS2	CNKSR3	AP1S1	ABI2	ARNT2		
PKNOX1	DNAJC5G	USP8	EIF1	ZNF652		
MRS2	ARL10	EIF3J	LANCL1	ZNF507		
TUBGCP3	JAZF1	LRRC28	CAPZA2	ZHX3		
PPIL6	SPDYE1	UTP15	POLD3	SAMHD1		
TMEM179	GATC	FAM234A	DBF4	OPA1		
RNF217	TUB	OPRM1	FAF1	FAM169A		
CIITA	TMEM26	FAM168A	PXMP4	YPEL5		
RNGTT	CMTM4	TAF1	BRCA1	DNAJC27		
KIAA0513	SRPK2	DTX3	PGLS	SHC3		
RNPS1	SP8	VTA1	NUPR1	ADD2		
SYNPO2	MACC1	TRIM40	EPN1	TMEM127		
NTMT1	ATF4	MON1B	TRHDE	PRR11		
FARP1	PXDC1	MLLT3	PNISR	RSBN1		
FSD1L	CREB3L2	ACSL1	CX3CR1	STRBP		
XKR9	NAIF1	BORA	SMIM10L2B	PAG1		
IQCE	RINL	CRISPLD1	SPDYE11	ERMAP		
CLTC	APOOL	PCMTD1	SPDYE17	KLHL7		
ELP4	LONRF2	CBR1	C4orf54	WDR5B		
DNM1	CIBAR2	CNOT6L	ATP2B1	ADCY2		
ABCA8	NHLRC1	DCAF10	PTAR1	PDP2		
PLAGL1	SIAH3	PTTG1IP	SPDYE14	ZBTB4		
FAM104A	TPD52L2	HECW1	SPDYE8	EPB41L5		
PRMT7	MOB4	KIF17	SPDYE15	ADCY1		
CEACAM7	GPX1	JADE1	SPDYE9	INIP		
IGF1R	CLEC12B	COL4A6	SPDYE13	XYLT1		
WASF3	GADL1	CYFIP1	MCTP2	LMBR1		
CDC27	NEBL	SNTG1	IMPG1	HHIP		
CCR1	YKT6	TMEM9	CASR	HAPLN4		
MIER3	ETV1	CST3	CD44	KLHL18		
CCDC125	ARFGEF2	CSF1R	REEP3	VCPIP1		
C1orf43	ANO6	DBNDD1	OR4N5	HAPLN1		
MAMSTR	PAFAH2	TMEM273	OR6B1	AFF2		
ZNF546	PPP6R3	VSTM1	BIRC5	FNTA		
TSEN15	KLHDC2	RMND5B	SOGA3	KLRD1		
LYPLAL1	LOC102723728	ZNF585A	KATNAL1	PRKAR2B		
SYT7	TXN2	LAIR1	MECR	PRPS1		
CDK19	GPR55	DLD	CSKMT	QSOX1		
EYA4	KCNK9	ZNF761	TF	KCNK5		
TRPM7	KIAA1958	UHRF1	ATG9A	BMPR1A		
HMGB3	PGAP2	SEMA3B	RAP1GAP2	SPTLC2		
DEFB134	NWD1	TIMM23B	PAK3	IDE		
TMEM198	COL20A1	MYADM	SLC2A5	SMAD4		
HAUS3	SLC30A3	CDIN1	CCDC85C	INHBC		
PRKACA	ITPR1	SLC35E2B	PTPRQ	LPP		
ZBTB26	SAV1	ZNF304	ANKRD34C	DCAF7		
CEP20	WNT10B	CYB561D2	ANKRD33B	PITPNA		
PRKAG2	TUT4	SH2B3	TK2	UQCRB		
GPX8	POTEB3	ZBTB8A	CXADR	SEC24B		
SERPINB13	MTMR9	REL		RAB35		
MAP4K2	TTC34	KLRF1		SLC2A3		
ELMOD1	SNX27	MTR		ZNF81		
TNS1	CEBPZOS	CDH18		PHLDA1		
SENP5	PLEKHM3	RUFY3		PITPNB		
NEURL1B	ING5	SGK1		WBP2		
CPEB4	TMCC1	CLNS1A		DSE		
GCLM	TPRG1	MYCN		MCTS1		
PGPEP1	SH3RF2	CA12		DBNL		
SLC38A7	HEY2	SEPTIN9		HDAC9		
THY1	HNF4G	LYRM7		CTSO		
BLOC1S6	HPSE2	ZNF131		ARL17B		
RHOA	SCAPER	HSPBP1		KCNJ5		
LOX	PLEKHJ1	CNGA3		PPT1		
ZNF331	PRIMA1	CIRBP		VMA21		
TRAM1	ATP2B3	FRMD8		SLFN12L		
PPM1L	SLC4A4	ZNF562		LRCH3		
ZKSCAN5	SLC6A11	KMT5B		OTUD4		
LZTS2	SLC25A15	EMSY		ZNF577		
NINL	TENM1	DLG2		MAPKAPK5	
CCDC157	NPTXR	NUDT4		ADPRHL1		
ZFP1	SMUG1	CADM1		NBEAL1		
TRAPPC4	CRCP	NDST1		ZNF142		
STK32C	GOLIM4	ZBTB44		SPECC1		
OPCML	HERPUD1	ERC1		RNASE10		
RILPL1	EEF1AKMT4-ECE2	TMEM98		SFMBT2		
CAMKV	DAZAP2	UMAD1		OR10A6		
NRXN1	TSC22D2	AAMP		BMP7		
U2SURP	TRIL	ACHE		SLC26A4		
FAS	TECPR2	CEP97		GABRA1		
BNIP2	PPM1E	SMIM21		GUCY1A2		
LPGAT1	RRP1B	VBP1		HTR4		
MCF2L	WDR43	CD226		OPRD1		
WDR82	OTUD3	OLFM2		PTGIS		
TTYH3	KIF13B	LMOD3		IL17REL		
NDFIP1	VPS39	HMG20A		OR8D1		
LRRC27	ANGEL1	KRCC1		SPIN3		
DDHD1	AHCYL2	CYREN		FAM102B		
CYRIA	TMEM158	ZNF236		SNX30		
TXNDC5	UBXN7	QDPR		SERF2		
APOLD1	GPX7	DAPP1		YTHDC1		
OR2J2	MED15	CCT5		HIGD1A		
ANP32E	PHF20L1	TGFBR1		VSIG4		
AMMECR1L	RTRAF	TCF12		AUTS2		
TRAPPC9	IRAK4	ZYG11A		AP4S1		
MARVELD1	A4GNT	UPRT		ARHGEF3		
CACNG8	NCKIPSD	DYNAP		CALU		
CCNB1	PAX5	ST8SIA5		KCTD1		
PCDHGA6	TRIT1	LIPG		NEMP2		
CAPRIN2	TENT5C	PIK3C3		SIN3A		
PLXNA1	THUMPD1	PHC3		PDCD6IP		
EIF1AD	HIF1AN	ZNF24		STX3		
LLPH	SLC30A6	SYT17		SRSF10		
DDI2	POLR3B	SS18		TMEM178B	
COG8	EFHC1	SSR3		RUNX1T1		
MSANTD4	GOLPH3L	ZNF346		SLC28A3		
JAGN1	PARVA	NME6		TMEM235		
KRTAP4-4	SLC47A1	NAA50		TAF1C		
TRIM63	UQCC1	MGME1				
PAPOLA	CMTR2	AURKB				
MAFG	MINDY1	MYMX				
SFT2D3	CHST11	CCDC141				
ZC3H10	GABRQ	SPC24				
SYAP1	DISC1	BLCAP				
SPPL2A	WDR4	CTSB				
ITIH5	ERBIN	ARIH2				
SPRYD3	FBXO42	CDKN2AIP				
PRPF38A	ANKIB1	THAP6				
TMEM241	WDR44	BCL2L1				
KRTAP4-11	VSIG10	AQP7				
SYNE1	CHST7	SLC35E4				
ZCCHC3	HMCES	ZNF532				
SLC45A3	STOX2	BICRAL				
PML	CDC42SE1	FHL2				
SMDT1	CLDN2	LSAMP				
CDC14B	JPH2	FAM216B				
CFAP91	AICDA	PRCP				
MVB12B	SERINC1	NUMB				
DISP2	TBC1D14	BCAS3				
TPD52L3	ZBTB2	DMAC2				
FGF13	TRPM3	MED28				
CDH7	BMP5	KDM7A				
CORO2A	MTMR3	ATXN3				
WDR7	CLDN1	OR2H1				
AZIN2	DMTF1	C1QTNF3				
ZFP91	PSAT1	PHACTR1				
MYLK	ADAM22	ADAMTS12			
TEKT1	FOXA2	SFXN3				
BSND	OPTN	SLC25A28				
FBXO32	PRSS22	CDK13				
SLC36A1	IPPK	DNAL1				
COL9A1	DDX50	CRISPLD2				
PTPN2	IRF2BPL	GRWD1				
C20orf96	RPAP2	CACNG6				
CST9L	PRR5L	ESYT3				
SRXN1	CNR2	KLF16				
WFDC10A	COL4A1	PCBP2				
ASB16	CTSG	TMEM245				
COLEC12	FCN1	ZIC4				
DPP8	HOXD10	ZNRF3				
UBE3B	LY75	ZNRF1				
INPP5K	MN1	LRRC8C				
ZFP82	NMBR	HSDL2				
HPCAL1	OMG	CCDC77				
PANK1	PFDN5	ARFGAP2				
GLB1L2	PIM1	MEGF11				
ZNF251	PREP	SPIRE2				
MARCHF9	MAPK13	CNFN				
CMTM7	PSMB2	KBTBD8				
ARL11	SCN9A	BOK				
DDX31	ITSN1	NT5C1A				
BCL2L11	SH3GL2	ST6GAL2				
NFAT5	SLC7A2	ZNF594				
SPATA17	SPARC	SLITRK2				
VANGL1	MAP3K7	USP38				
NRXN3	VCL	MFSD14B				
SPPL3	ZNF84	INA				
MAPK9	HMGA2	TXNDC17				
SREK1	TAF15	ZNF496				
DNAAF8	RANBP3	ADGRA2				
TSTD2	DGKE	ZNF514				
PPTC7	DENR	CEP89				
CLDN14	ASAP2	PDGFB				
TBC1D2B	ARHGEF7	TSLP				
MAPK1IP1L	MBD2	GTPBP10				
ATP6V1C2	EFNB2	NKD1				
ZFYVE27	COL12A1	CNP				
C15orf40	DMP1	GABRG3				
NIPA1	FOXE1	ADAM19				
CMTM3	ILF3	TNKS1BP1				
UROC1	RAD9A	DMAC1				
FAM76B	RAB11A	LARP1				
GLT1D1	VAPB	GADD45GIP1			
SEPTIN10	MAGI1	RFT1				
KLHL23	IER2	SLC2A13				
EFHB	DHX8	ELFN2				
SPICE1	SERPINI1	WDFY2				
FAM91A1	CD80	NIBAN1				
FAM124A	CDX4	ARTN				
UBQLNL	SKIL	KLHDC3				
C18orf25	ARHGAP6	NAA15				
PIGM	NCOR2	TRIM6				
DIRAS1	SPTLC1	BORCS5				
AFG1L	ELL	BRD4				
PDCD4	RRAGA	BANP				
WFDC5	TCFL5	B3GALT6				
APOBEC3A	LMO4	TMEM41A				
TIGD1	HNMT	SLC46A1				
CACNG5	SRP72	DYNLL2				
HS6ST2	WASF2	SIRPA				
FOXP2	ZHX1	SOCS4				
OSBPL9	DUSP12	MUCL3				
RPUSD2	KLF12	DBN1				
C1orf158	DNPEP	WRNIP1				
C1orf216	GTF3C4	TSPAN18				
JMY	MRAS	SYCE1				
APOBEC3D	KPNA6	SNAP25				
CEP128	SEC61A1	WIPF2				
ZNF362	SNX12	GSX2				
SNRNP48	NRBP1	PLCD3				
TBCEL	OSTM1	CCBE1				
ELAPOR2	TRPS1	BMPER				
SCUBE3	HNRNPH3	MED22				
TMEM18	UROS	RORA				
TMEM268	PISD	ZNF845				
TNPO1	NEK9	DHRS1				
MFSD4B	METTL21A	FOXP4				
ODF2	CCDC40	TLR4				
LTO1	CEP78	MPLKIP				
HS6ST3	ZNF383	CANT1				
CREBRF	FBLIM1	ANKRD54				
CALHM5	MGAT4C	A1CF				
C10orf67	ZNF843	MS4A6E				
MEIS2	GCK	KCNH5				
SKP1	NT5C3A	YWHAB				
CAMK2B	DCHS2	ODAD1				
CAMK2G	LOC102723996	NACC2				
ELF1	DEPDC4	ZNF558				
RELN	C9orf85	CCDC13				
IFNLR1	MIOS	KLK11				
ABCA12	LOC102724488	ZFC3H1				
NFATC3	TFDP2	VSTM4				
SLC38A11	GIGYF1	ANKRD23				
REM2	SH3D19	ZNF25				
CCDC171	NFILZ	GLYATL2				
RNF152	RNFT2	NSMCE1				
SLC9A9	SPDYE21	SENP8				
ZNF619	LCA5L	OSBPL3				
SLC30A8	OR6C4	DBF4B				
SPATA19	TTC28	TRAF6				
CNTN1	ESR2	CRYZL1				
KRT73	ING2	SNX15				
MT1E	NCF1	IPMK				
EXOC8	TPP1	TCEAL7				
C5orf51	NEU1	ORAI3				
PIGN	PCSK1	TADA2B				
PDLIM2	SLC10A2	RAB42				
PATJ	CYP3A7	TRMT61A				
STX12	GRIN2B	PIK3AP1				
LYNX1	IGF2R	AGBL1				
RETREG3	TSR3	CDYL2				
RASSF3	NOMO3	PRXL2B				
SPTBN1	OR2T33	TBX15				
LCE1E	OR5A1	TMEM237				
SPINT4	OR56A4	UGT3A1				
SLC25A42	OR56B1	C5orf24				
TRIML1	OR4N4	ZNF664				
FBXO27	IGSF3	UBE2U				
SCYL3	GP2	CNIH3				
DCAF4	CISD2	ARL6IP6				
NCOA3	PLPPR5	PPM1K				
GJA5	DNTT	MOSPD2				
NF2	DAPL1	SDE2				
ZSCAN22	CORO1B	BBS12				
APAF1	ZNF181	SGMS2				
ENOX2	AP1G1	CDC20B				
GATD1	CYB5RL	DCP2				
TMEM255B	MAF	SNX31				
ANKDD1A	NGRN	DAND5				
TPRG1L	ELMO1	NANP				
VSX2	DEDD	AMER2				
ERG	MINDY2	MTURN				
IRF2BP2	SLC6A3	COL22A1				
DTNBP1	JADE3	MAGI3				
TMEM107	G6PC2	PXT1				
CYP26C1	POLR2J3	ZFP3				
HNRNPA3	MID1IP1	SLC35G1				
ZBTB41	GXYLT1	ERI1				
RNF41	PACS2	APCDD1L				
RNF138	KIF13A	ZCCHC24				
TREML4	SYNPO	B4GALNT2				
G3BP1	IGF1	MED19				
ZNF621	PABPC4L	NUP50				
FAM83H	PLAAT3	LCORL				
NHLRC2	ROBO2	CACUL1				
CCDC172	INSYN2B	ZFPM1				
DRAXIN	MEF2A	ADGRF1				
LHFPL4	AGFG1	RASGRP4				
TDRD1	PIK3IP1	ATP2A2				
BCAS4	RPS24	RASAL2				
ACACA	ZNF880	NMNAT2				
LCN6	SPDYE6	CAMK2A				
DZIP1	CCDC81	TRIM39				
ISM2	PLEKHD1	RC3H1				
ILDR2	COG5	CSF1				
KCNRG	ZSCAN12	MMAA				
CACNB2	C10orf105	KCNH1				
ZMYM3	SPDYE2B	LIN9				
ENOSF1	NEU4	USH1G				
SMIM10L2A	UBAP1	PTCHD1				
SYNJ1	ASTN2	TTBK2				
GRB2	FBXL20	PLBD2				
UGT1A6	CENPO	KLHDC8B				
ECI2	KATNA1	TYSND1				
PRAME	ATG13	ANKRD52				
ENSA	EHF	ZNF776				
FREM2	BACE1	RIMKLA				
LRRC75A	GHR	PDZD8				
BEND4	PARN	RABL3				
TMPRSS11F	GFOD2	SLC41A1				
TEX19	CDC5L	PSMD12				
ADAMTSL3	NKX3-1	CCDC50				
ZC3H14		SEC14L3				
EEF1AKMT2		ZPLD1				
ANO5		DDX51				
TMED8		APTX				
WNK1		XG				
ANKRD44		PTPMT1				
KDM2B		ZNF792				
PEAK1		PRR18				
PCGF2		FGF14				
RECQL5		PSMD13				
RAE1		FAM171B				
METTL6		PKHD1L1				
UBE2E1		ASB6				
SACS		SOX5				
SNX9		GPR119				
SYNJ2		CCDC190				
ARHGAP5		ZDHHC21				
DRAM1		IQUB				
MICU1		HPS5				
ADAR		PIK3R1				
SUSD4		UBXN2A				
FOSL2		OPN5				
CHIC2		CERS2				
TRIM2		C20orf203				
DELE1		SKA2				
MYLK4		CTDSP1				
EMP2		GPX6				
ACTN4		GDF7				
RPS6KA3		DLGAP4				
DLG3		ADCY5				
PHKA1		RNF14				
AGPS		FBXO25				
LOC401040		SRRM4				
TTC23L		KRIT1				
MKRN1		COPS8				
STX17		CD96				
NIPSNAP3B		MLX				
PLA2G15		HIPK1				
TMEM131L		SRC				
NR3C2		EMB				
ARHGAP25		C19orf54				
GALNT16		RUFY4				
RIMBP2		LRIT3				
DMBT1		LANCL3				
INPP5F		STIMATE				
ZC3H12C		GJB7				
HMGCR		VWC2				
KAZN		SULF2				
CACYBP		KRTAP10-4			
NFIA		KRTAP10-5			
TMEM234		MKNK2				
EFCAB2		HOMER2				
ABCC5		POLQ				
SNCA		CDC25A				
SUPT3H		MAFA				
EXOSC2		STAMBP				
RAPGEF3		SF1				
TARBP2		IYD				
GRTP1		MBLAC2				
NTRK3		IER5L				
TVP23A		OR2V2				
TTLL6		NAALADL2			
SOCS7		PAOX				
NPEPPS		FAM199X				
GOSR2		NIPAL1				
ZC3H4		SPRYD4				
ARHGEF9		MAP6				
SHROOM4		PATE2				
AGK		SIGLEC15				
MLLT10		SLC25A24				
ENAH		PRRC2C				
EIF5A		NYAP2				
TCEA2		USP37				
HECW2		CDK15				
RBM5		HSPBAP1				
LIMD1		PI4K2B				
B3GNT3		ABI1				
UQCRQ		SLC1A2				
MOCS3		IDH3A				
MCAT		GLP2R				
OR1A1		NKIRAS2				
STRN3		RARA				
DNTTIP2		FOSB				
FAM120A		ZNF324B				
NOS1AP		PDCD5				
TCAF1		GMEB2				
KIAA0586		CRELD2				
TOMM20		CHCHD5				
LCMT2		PDS5B				
TOX4		SLC66A2				
RHOBTB1		ACAD8				
SRGAP3		FAM172A				
HELZ		KIAA0319L				
NLGN4Y		MEF2D				
NAV3		STAT1				
CNKSR2		TTC21B				
FASTKD2		SNCB				
STK38L		MAFK				
TNIK		CACNA1C				
GGA2		LRP6				
SPART		SETD1B				
TTLL12		LITAF				
SEPTIN8		CCNP				
RCOR1		PGLYRP4				
CYLD		ADAM23				
CUX2		GABBR1				
HAUS5		GRB10				
RRP8		CEP152				
BRI3		HACD3				
CCDC28A		MPHOSPH6			
C2CD2		PAFAH1B1				
CRELD1		POU2F2				
DNM3		THOP1				
LSM14A		TNNC2				
FAM184B		PPM1F				
WWC3		BIRC6				
RRP7A		ACTR8				
COL5A3		PDE10A				
KRT76		ATG14				
ST8SIA3		POMT2				
ZMYND10		SRGAP1				
RWDD1		CEP290				
GAL		ZDHHC6				
ABHD5		SPOCK2				
DHRS7		ZMYM1				
RLIM		GNG4				
RAB10		BIN3				
MRTO4		PPIH				
HECA		CHRM3				
DCTN4		PODN				
CYB5R4		NPHP4				
NLK		TLR5				
AKAP11		CYP27A1				
CAB39		EAF2				
WBP11		TGFBR2				
GULP1		BAP1				
RAPGEFL1		ADGRL3				
ZDHHC2		LARP1B				
PHF20		OGFRL1				
BET1L		AGFG2				
UFM1		FGFR1				
CYRIB		TUSC3				
C21orf91		ANKS6				
EPHB2		SNAPC3				
BAIAP2		ASAH2				
NPTN		CPT1A				
PLXNA3		RBMS2				
LEPROT		DCAF5				
PARP14		PNPO				
TRIM44		SMIM5				
DIRAS2		PIGL				
KIF21B		ABHD12				
ZNF280D		TAB3				
GDAP2		GALNT11				
LRRFIP2		ASPH				
UHRF1BP1		CC2D2B				
CASZ1		RPS6KA1				
NSD3		CNTN2				
DEPDC1		TNRC6C				
SARS2		TXNL1				
PIGX		IL18R1				
GID8		TIGIT				
TTC38		UBL4A				
VPS37C		CLEC4M				
TMEM248		ADAM28				
IFT57		CAPN6				
CCDC186		TNFAIP8				
PRPF38B		SND1				
ANO10		TMOD2				
ARMC1		TMEM97				
KLHL11		SOX8				
N4BP2		HERC3				
SBNO1		KIAA0040				
ARL8B		PHF14				
MACO1		EDEM1				
MOB1A		AQR				
RCOR3		SECISBP2L				
TMEM19		ZNF536				
C5orf22		RNF144A				
AGPAT5		SERTAD2				
LIN7C		EPM2AIP1				
SYNJ2BP		PLPPR4				
BEX1		KBTBD11				
GPALPP1		ADNP2				
CHST12		CILK1				
RBBP6		ADGRL1				
CTTNBP2NL		ZNF365				
SLC30A10		RPH3A				
OTULINL		SV2C				
SPATA6		TNRC6B				
UGT1A10		RAD54L2				
UGT1A8		PHF3				
UGT1A7		LARP4B				
UGT1A5		TRIM9				
FGF20		FBXO28				
TCIM		MINAR1				
SAR1A		POFUT2				
RAD18		MGRN1				
CMC2		ADCY6				
NIT2		SASH1				
OTUD7B		DPY19L1				
DTWD1		ESYT1				
CDC42SE2		PHF24				
TULP4		SRGAP2				
ARRB1		ZNF10				
MNT		SPEF1				
SLC39A10		CAMSAP1				
ENTPD7		ATL3				
XPNPEP1		NOL11				
PLXDC1		RAB11FIP5			
ADCK1		DKK3				
LYRM1		NUDT13				
SELENON		TRIM33				
NHSL1		TPPP3				
NDRG4		UBE2D4				
RHOJ		KLF13				
HEG1		FIS1				
AARS2		HDGFL3				
MIB1		INSIG2				
TRMT5		SUFU				
CCDC191		IKZF2				
ZFP28		AK3				
MEAK7		PCYOX1				
SLC7A14		ACP6				
LZTS1		ANKFY1				
UGT1A9		SCLY				
TMSB4X		KLF3				
RNASEL		ERGIC2				
NEUROD4		SLC25A37				
NTN4		ZNF771				
RBM25		FZD3				
BCR		SIX4				
PBOV1		SPA17				
ADAM12		CPSF2				
FAM3A		NAGK				
TWNK		OTUD5				
MCL1		SAMD9				
ZNF148		GIPC2				
LAMTOR3		FERMT1				
KCNK13		NUP62CL				
KCNK12		BIVM				
NAPB		CWC25				
CLSPN		RPP25				
TSPYL2		NHP2				
CLSTN2		PHIP				
RAB38		ZNHIT6				
PDF		ARGLU1				
NCAPG		ATG2B				
ABCG8		MANSC1				
NSD1		NODAL				
GREM2		RIC8B				
ATPAF1		CCDC198				
CERK		CKAP2				
NIBAN2		UBA6				
BCL11B		BRF2				
C8orf33		SPTLC3				
FUNDC2		SLC39A9				
C1orf116		FOXJ2				
TMPRSS3		TMEM242				
PRRG3		ASAP1				
MBOAT7		ZC4H2				
TMEM43		CEP41				
DSC3		AJAP1				
ADIPOR2		PCDHB14				
GALNT14		PCDHB15				
EFCAB1		PDE7B				
TUT7		ERRFI1				
RTL10		HOXC5				
VPS37B		SSH1				
SNIP1		CCSER2				
IQCA1		TMX3				
MOB3B		ING3				
ATP8B4		SEPTIN3				
ACTR5		PPP1R37				
FBXO17		HOXD8				
POF1B		LRRC8A				
DOCK5		C21orf62				
GFM1		SERTAD4				
ASRGL1		ACRV1				
NYNRIN		FEM1C				
CENPA		C5orf15				
CKS1B		INCENP				
SLC31A1		COQ8A				
DR1		ZMIZ1				
DRP2		SLC24A2				
DSG2		PCNP				
FGF4		LYZL6				
FRK		THAP11				
GABRR2		RTN4				
GFPT1		RCN3				
GLUL		KIAA1143				
GNAQ		GATAD2B				
GTF2E2		LRRC47				
GTF2F1		USP31				
KDR		NLN				
LAMA4		CCPG1				
LAMC1		NLGN4X				
LIFR		MTUS1				
NDUFA4		COG6				
NEO1		PCDH19				
NPTX1		RTL9				
OR1D2		NUFIP2				
OR3A1		IFT80				
PPP2R1B		RELCH				
PRPSAP1		SHROOM3				
PSMD7		CALCOCO1			
PTPN3		PLXNA4				
RAP2B		GNAO1				
RBL1		ZNF253				
RGS12		ZNF250				
RYK		ART4				
SBF1		CCAR2				
SH3BP2		LY6G5B				
ST8SIA1		IL21				
SOX11		FGA				
SSR2		ITM2B				
SYN2		TIA1				
TAC1		NXF3				
NFE2L1		MRPL17				
TEF		VIPAS39				
TRPC1		STEEP1				
UBE2G1		EPB41L4A				
UBE2N		ERAP2				
VLDLR		NXN				
WEE1		SUDS3				
WNT2		VPS52				
ZNF177		GPR135				
SNN		PRRX1				
ULK1		SLC13A3				
VAPA		MRPS11				
GCM1		INTS3				
TRADD		ZBTB10				
CDS2		ZNF426				
CCN4		DSC2				
MBD4		PSTPIP2				
KYNU		WNT2B				
USP13		FYCO1				
INPP4A		NKAP				
EIF4EBP2		RIC3				
AKAP6		MPZL1				
ATF6B		MAP7D3				
ELF3		NAA35				
ETV5		YRDC				
FGF10		LRRK1				
VEGFD		ASB13				
FOXA3		TXNDC15				
MYT1		SNX22				
PEX14		L2HGDH				
GET1		SCD5				
COIL		NAA25				
LIN7A		IFT74				
DCLK1		CLU				
PIWIL1		GOT1				
B4GALT5		HOXB5				
UBE4A		IDO1				
KCNK6		IFNA8				
SH3BP5		LRPAP1				
PCYT1B		LSS				
AKAP5		SH2D1A				
SEC22B		MARCKS				
MYO1E		MCC				
NDUFA9		MSR1				
NFKBIL1		NFX1				
SNU13		NOVA2				
OXA1L		OAS1				
SCD		PAPPA				
ZMYM4		PBX2				
ADAMTS4		PDCD2				
THRAP3		PDGFRB				
CLEC2B		PDK2				
FOXL1		PLN				
GPR15		POLA2				
HAS2		PPP2CA				
PRKAB2		PPP6C				
CCL13		PRKACG				
AKT3		PRKCB				
MLANA		PSMA4				
HOXA11		PTMA				
IFNGR2		PTPN4				
LAMC2		PTPRG				
SOX3		RAC2				
VAMP7		CCL22				
FARSB		XCL1				
ABCC4		SDC4				
SAP18		SFRP4				
GOLGA3		SHB				
MAP1B		SLC6A1				
CD164		SLC18A2				
ALKBH1		SSR1				
HTR3B		SUPT4H1				
HS3ST3B1		TFAP2B				
CITED2		TRAPPC10				
NTS		TNNC1				
ORC2		TRHR				
PAX9		TXK				
ENPP1		UBE2B				
PHKG1		UBE2V2				
PRKAA2		WNT5A				
NCOR1		ZIC1				
RBM14		ZNF141				
TRIM38		BSN				
EIF3M		BFSP2				
ARL6IP5		CUL4A				
MTHFS		TPST2				
PAICS		SMARCA5				
P3H4		GPR65				
POLR3F		PARG				
GNAI3		NIPSNAP1				
HIC1		TP63				
PVR		AOC3				
HBS1L		CHRD				
MTHFD2		IRS2				
MMP24		KAT2B				
NUDT3		ST13				
SUGT1		RNF8				
DNAJB2		FKBP5				
AP3M2		GMFB				
CKAP4		SLC1A1				
TMED10		SNRPD3				
RAB31		USP2				
ZFHX3		CHST2				
IDH3B		GDA				
RASGRF2		ARHGDIA				
DDX52		ELAVL2				
ADAMTS5		ETFDH				
NISCH		FOXD2				
CEP250		HNRNPC				
SEC23IP		SCO1				
RNF139		PIAS2				
KLHL2		RABEP1				
MME		ATG12				
BRD3		RPS6KA5				
ATP1B4		ASIC3				
ELL2		B4GALT6				
CHRM5		BAG5				
CLDN12		APBA3				
TIMM10B		CDH6				
KCND2		DOCK3				
LDOC1		GABRE				
MYCBP		KCNC3				
PRND		KCND3				
POLM		NUCB2				
CDIP1		ABCD4				
OR10H1		TLE1				
PAX8		AKAP17A				
CNTNAP2		EXOG				
PRKAR2A		ABL1				
PMS2		ATP5MC2				
NUTF2		CBL				
CDKL5		CBX2				
ZNF197		FOXN3				
ZEB1		CRK				
CSTF1		CRKL				
HMBOX1		ERBB4				
LRP10		HSPA1A				
PLEKHA7		CNOT9				
SRGAP2C		ZRANB2				
ASB1		KCNE3				
MOK		CFL1				
TIMELESS		ITK				
POLA1		KRT6B				
CYB561		TAF11				
POU6F1		VDAC3				
AKT2		RNF103				
LRRC7		TSPAN5				
CMC1		CNIH1				
TMEM72		SPRY3				
CEP83		MLLT6				
DLGAP2		MMP16				
DYRK1A		MTF1				
AR		HDAC4				
ZNF525		PRICKLE3				
TBC1D5		NRGN				
HSPB7		PRKG1				
ICA1		DNAJC3				
SLC8A1		HOXB13				
C13orf42		VTI1B				
DAZAP1		CRTAP				
CTXND1		AGPAT1				
PSME3IP1		TGOLN2				
BRAF		ERF				
BRD3OS		PSMC4				
EPM2A		TRIM27				
DIO3		DYNLT3				
TCAF2		EXOC5				
PDE1A		DCTN6				
NR3C1		FUT9				
MGAT1		KIF1C				
FAM243B		HPSE				
TENT4B		SPIN1				
KLHL33		ABLIM1				
DAB1		FKTN				
GPRC5C		SCML1				
KPNA5		KRT38				
CACNA2D1		ZBTB33				
FAT3		ATF7				
ALDH5A1		ZFP36L2				
FGFRL1		CALM1				
AKAP12		ATE1				
POLR3G		KAT7				
CTXN2		ZXDA				
CRYBG1		ERG28				
FBXO41		RNF2				
TMEM217		PMF1				
DNASE1L2		ABL2				
STXBP1		PSEN1				
PNPLA1		AP4E1				
LOC100133315		DGAT1				
PDE8B		ADAT1				
XIAP		AP3M1				
IRS4		ARL5A				
HNRNPCL3		DDAH1				
PRRC2B		CCNDBP1				
TDRP		DNAI1				
AGTR2		FBXL3				
DTNA		FBXW2				
USP54		FBXO7				
ELAVL1		TRIM32				
GATM		MLYCD				
HSBP1		OGA				
ID4		SLC35A3				
IFRD1		MORF4L2				
CEACAM1		TRAM2				
BTG1		OR2A5				
COL4A4		PITPNC1				
ERCC6		TINF2				
SHH		YWHAG				
ITGB3		DNAJC15				
JAK3		VPS4A				
KCNA1		RBM15B				
PRNP		EEF2K				
SOX9		BLNK				
TGFBI		SNX8				
THBD		PCDHB1				
TPM1		GREM1				
BLMH		RAX				
IL2RA		NRG1				
UGT1A1		LIG3				
EPX		CRIPT				
CRX		TOM1L1				
BCL2		FAM156B				
CHRNB2		CD99				
GRM6		ASAH2B				
HTR1B		NDUFA10				
IL2RB		ADAL				
KCNJ2		PYCR3				
OPRL1		CALM3				
PRRG1		CTF1				
RPL11		NDEL1				
RPL28		DRG2				
OR2A14		SCYL2				
PIM3		GLDN				
ASTL		SAP130				
ERICH3		SMYD1				
STUM		L3MBTL4				
DUSP29		USB1				
BMF		TOP2B				
NRARP		KLHL26				
OR5M3		ZNF287				
GBA		OR2J1				
ZNF780B		CHD1L				
PLA2R1		ZDHHC24				
LMBRD2		KIDINS220				
NEXMIF		GRAMD2B				
USP20		DGKB				
DNAAF9		NT5C2				
C1QL3		TIAM1				
GOLGA7B		PCDH15				
SLC9A4		TMEM269				
CREB5		ZNF214				
OLA1		CXCL8				
KRTAP5-10		NUDT4B				
PABPC1L2A		GOLGA8T				
PLIN5		CCDC163				
DGKK		RNF227				
CARS1		FRRS1				
LGR6		NAGA				
BCL7A		CYP4A11				
YIPF5		RAB41				
CD34		NOTCH2NLB			
SLC35F1		NOTCH2NLC			
RGS7BP		BAALC				
MTUS2		DLG1				
ZNF704		RGMB				
ARL4A		TTC8				
FOXK1		BCLAF3				
TRIM71		MAGT1				
TESMIN		ZNF479				
TYW5		ZBTB3				
RSPO4		ORMDL1				
TMEM70		GABRB2				
LINS1		HP1BP3				
PABPC1L2B		IKZF5				
INSC		GLI2				
EIF4G2		LRIG1				
TTC14		CNGA1				
ZNF391		BDKRB2				
UBXN2B		CHML				
FAM110C		ATXN7L3				
LDB3		DMAC2L				
CCDC88C		FUT1				
MYBL1		GPRIN2				
EPHA6		OR7E24				
CACTIN		KIAA1671				
TMEM120B		DPYSL3				
ZNF112		FXYD3				
ARSA		IRGQ				
ZNF207		OR56A1				
RNF169		HTT				
TIFAB		ERN1				
SH3RF3		GPC4				
ZNF578		GDI1				
PIP4P1		GSTA4				
CNPY1		MR1				
RPP30		ACTA2				
PCMTD2		AHR				
SRRM3		ARCN1				
PRH2		ARSD				
TENT2		ATP2B4				
CFLAR		ATP6V1C1				
EIF4E		POLR3D				
ZNF720		CCND2				
AKR1C2		CD38				
PAX7		ENTPD1				
SMIM13		BCHE				
ANXA2		CD40LG				
ATXN7L3B		ERCC8				
VASH2		SLC26A2				
C1orf198		GNAT1				
CISD3		LIPA				
FAM13C		MYL3				
CROT		NDP				
ZNF534		PDHA1				
NSMAF		PGK1				
CCDC169		SERPINA1				
RFPL4A		PKP1				
CASTOR2		SLC5A1				
PPP1R3G		HOXA13				
INTS9		NOS1				
UBE2QL1		ADH6				
NONO		ALDH2				
SCRN1		ATP4B				
MTFR1		CHRNB1				
NCEH1		CYP1A2				
RBMXL1		CYP11A1				
STAU2		GLRB				
TLCD2		GRIN2A				
ATP5MGL		PTGFR				
ZNF268		NCCRP1				
TNFAIP8L1		ZNF429				
TRMT2B		SPOPL				
AMMECR1		ARHGEF37				
ZNF559-ZNF177		CLDN18				
CPEB2		RABL2B				
VIT		DYRK3				
SEMA3E		GRK6				
SLITRK4		ZNF774				
NIT1		KRTAP5-2				
GIMD1		OR1B1				
ATP6V1FNB		OR10H5				
EFCAB13		OR2L3				
CCDC179		OR4K17				
ARPC4-TTLL3		OR52E4				
PHOSPHO2-KLHL23		LRRC55				
ST20-MTHFS		OR10G3				
BMP2		PLCXD3				
BMP3		SLC25A25				
CTSV		WDR35				
ANKRD12		COA5				
ELOC		C1QL4				
SLC19A1		MXRA7				
MFAP3		SHE				
CD99L2		TTC7B				
ZNF322		ZC3H12B				
ELOVL5		MYPOP				
RANBP6		NUDT17				
ZNF124		OR2G6				
TRA2B		C2orf68				
TRDN		GDPGP1				

## Data Availability

The data analyzed in the present study are publicly available on the GEO data sets database. The data sets used and/or analysed during the current study are available from the corresponding author on reasonable request.
